# Supplementary material for: Nanodrug rescues liver fibrosis via synergistic therapy with H2O2 depletion and Saikosaponin b1 sustained release
Source: Commun Biol. 2023 Feb 16;6:184. doi: 10.1038/s42003-023-04473-2 (PMC9935535; doi:10.1038/s42003-023-04473-2)
Supplement: Supplementary file 2 — Supplementary Information-New [file 42003_2023_4473_MOESM2_ESM.pdf]

Supplementary Information

for

Nanodrug Rescues Liver Fibrosis via Synergistic Therapy with H_2O_2 Depletion and Saikosaponin b1 Sustained Release

Mengyun Peng[#,1], Meiyu Shao[#,1], Hongyan Dong[1], Xin Han[1], Min Hao[1], Qiao Yang[1], Qiang Lyu[1], Dongxin Tang[2], Zhe Shen[3], Kuilong Wang[1], Haodan Kuang[1], Gang Cao*[,1]

[1]School of Pharmacy, Zhejiang Chinese Medical University, Hangzhou, 310053, China.

[2]Department of Science and Education, The First Affiliated Hospital of Guiyang University of Chinese Medicine, Guiyang, 550001, China.

[3]Department of Gastroenterology, The First Affiliated Hospital, Zhejiang University School of Medicine, Hangzhou, 310003, China.

* Corresponding author. E-mail address: caogang33@163.com

[#] Mengyun Peng and Meiyu Shao contributed equally to this work

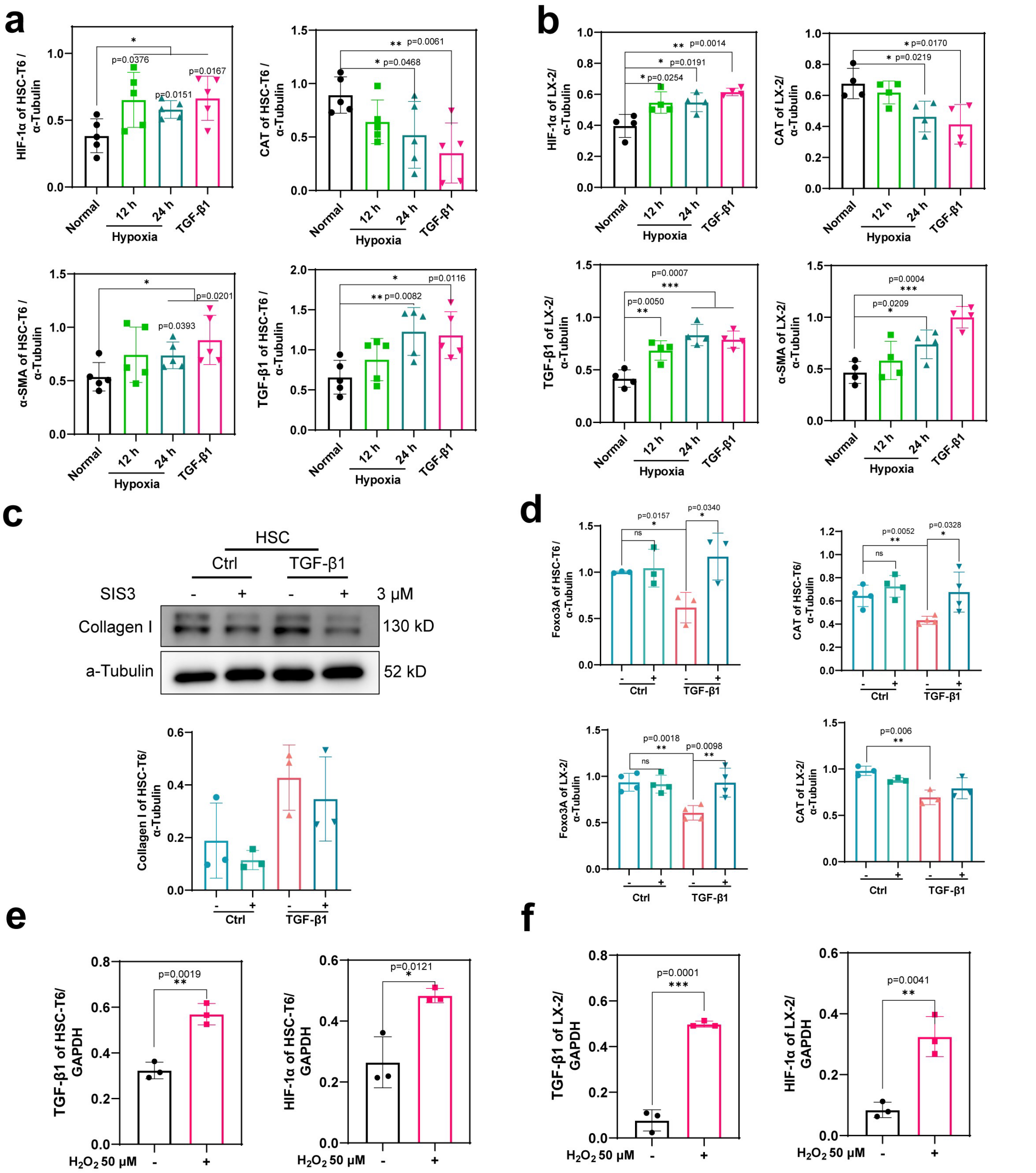

Supplementary Fig. 1. (a-b) Densitometric quantification of data shown in Fig. 2d (n ≥ 4, Mean ± S.D.). (c) HSC-T6 were stimulated with TGF-β1 10 ng/mL for 24 h in presence or absence of SIS3 at 3 μM and Collagen I was analyzed by western blotting (n = 3, Mean ± S.D.). (d-f) Quantification of data shown in Fig. 2e (d) and Fig. 2i (e, f). (n=3, Mean ± S.D., *P<0.05, **P<0.01, ***P<0.001 by Student's t test, compared to DMSO control)

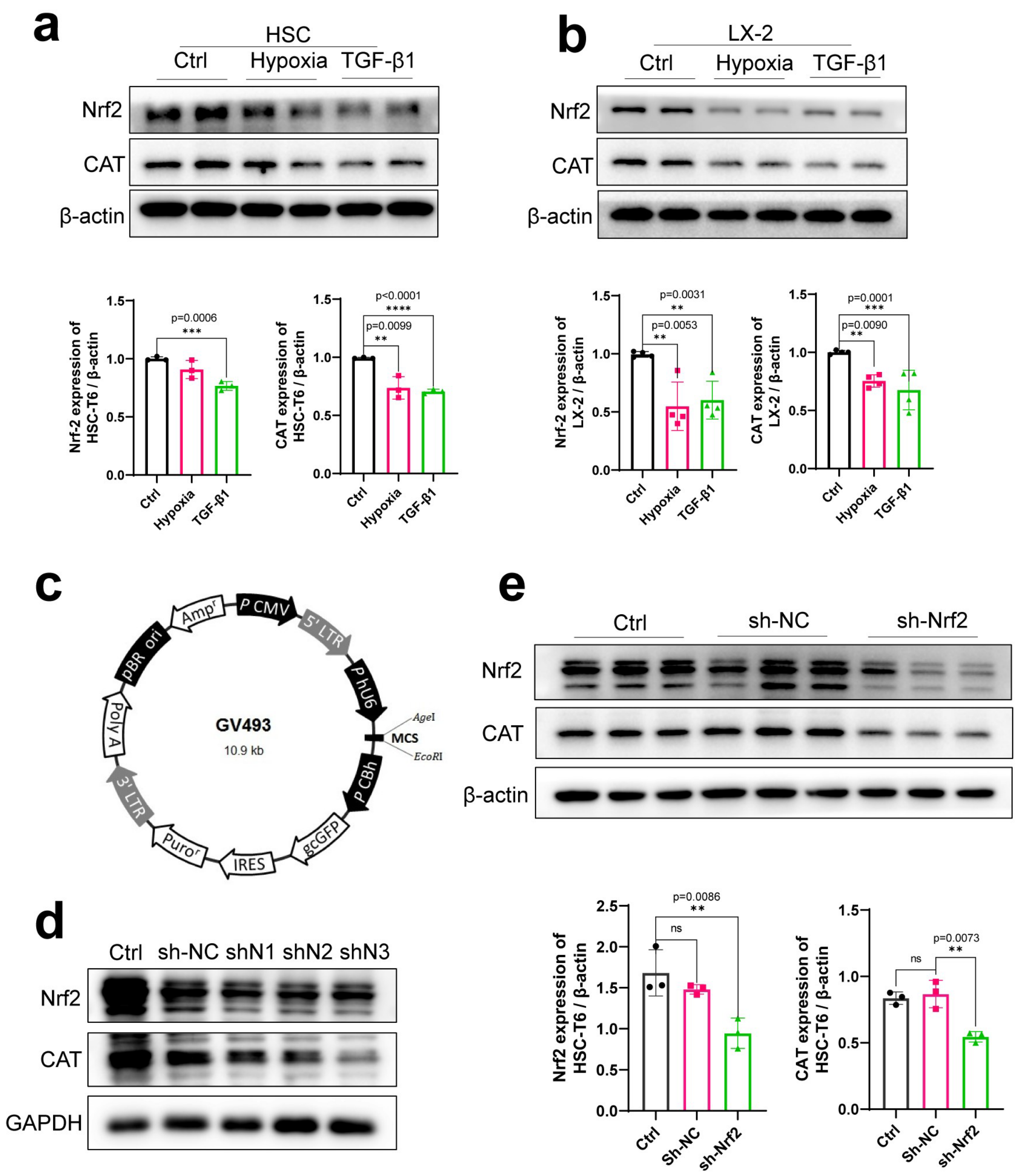

Supplementary Fig. 2. (a-d) Representative Western blots of Nrf2 and CAT under different treatment on HSC-T6 and LX-2 cells. (n = 3, Mean ± S.D., *p < 0.05, **p < 0.01; ***p < 0.001 by Student's t test.) (c-e) HSC-T6 cells were transfected with shRNA targeting Nrf2 or an empty plasmid as control for 24 h (n = 3, Mean ± S.D., *p < 0.05, **p < 0.01; ***p < 0.001 by Student's t test.). Protein lysates were prepared for immunoblot.

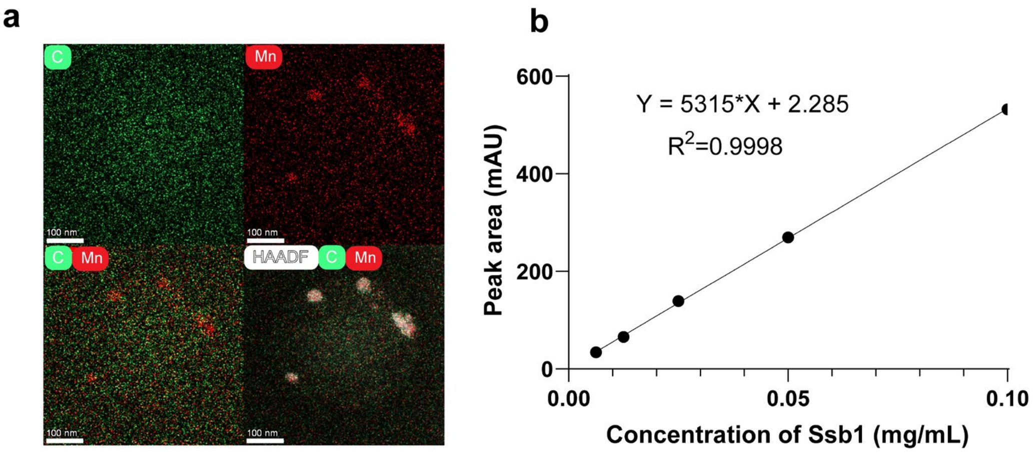

Supplementary Fig. 3. (a) Elemental mapping of MnO_2@PLGA/Ssb1 nanosystem. (b) HPLC standard curve of Ssb1.

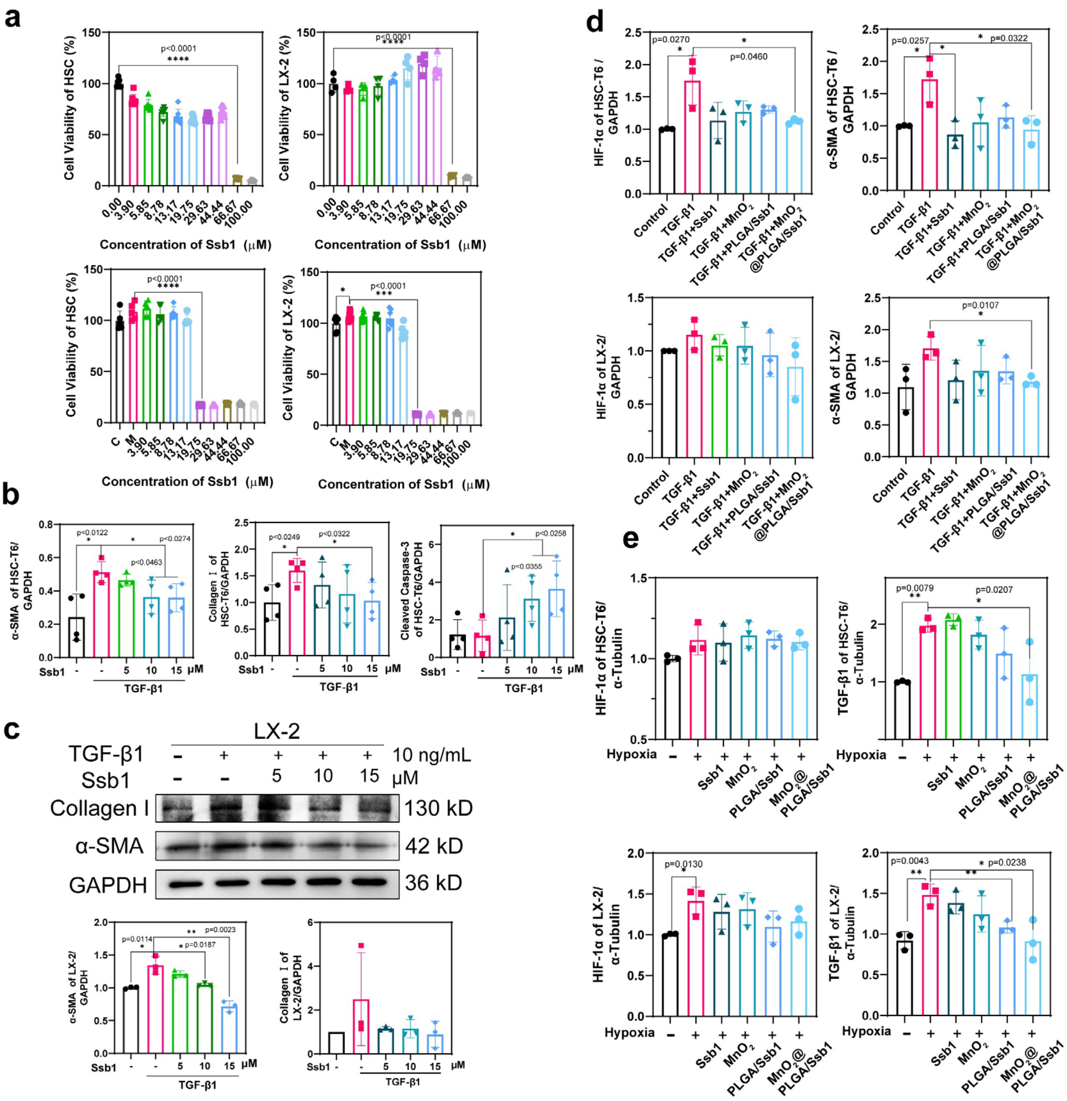

Supplementary Fig. 4. (a) Cell viability of quiescent and TGF-β1 activated HSC-T6/LX-2 cells with different concentration of Ssb1 (n= 4, Mean ± S.D.). (b) Densitometric quantification of data shown in Fig. 4a (n = 4, Mean ± S.D.). (c) LX-2 cells were exposed to TGF-β1 (10 ng/mL) and treated with Ssb1 (0-15 μM) for 24 h. Expression of Collagen I, α-SMA were determined by Western blot assay (n = 3, Mean ± S.D.). (d-e) Densitometric quantification of data shown in Fig. 4b-c (d), Fig. 4f-g (e), (n = 3, Mean ± S.D., *$p < 0.05$, **$p < 0.01$; ***$p < 0.001$ by Student's t test).

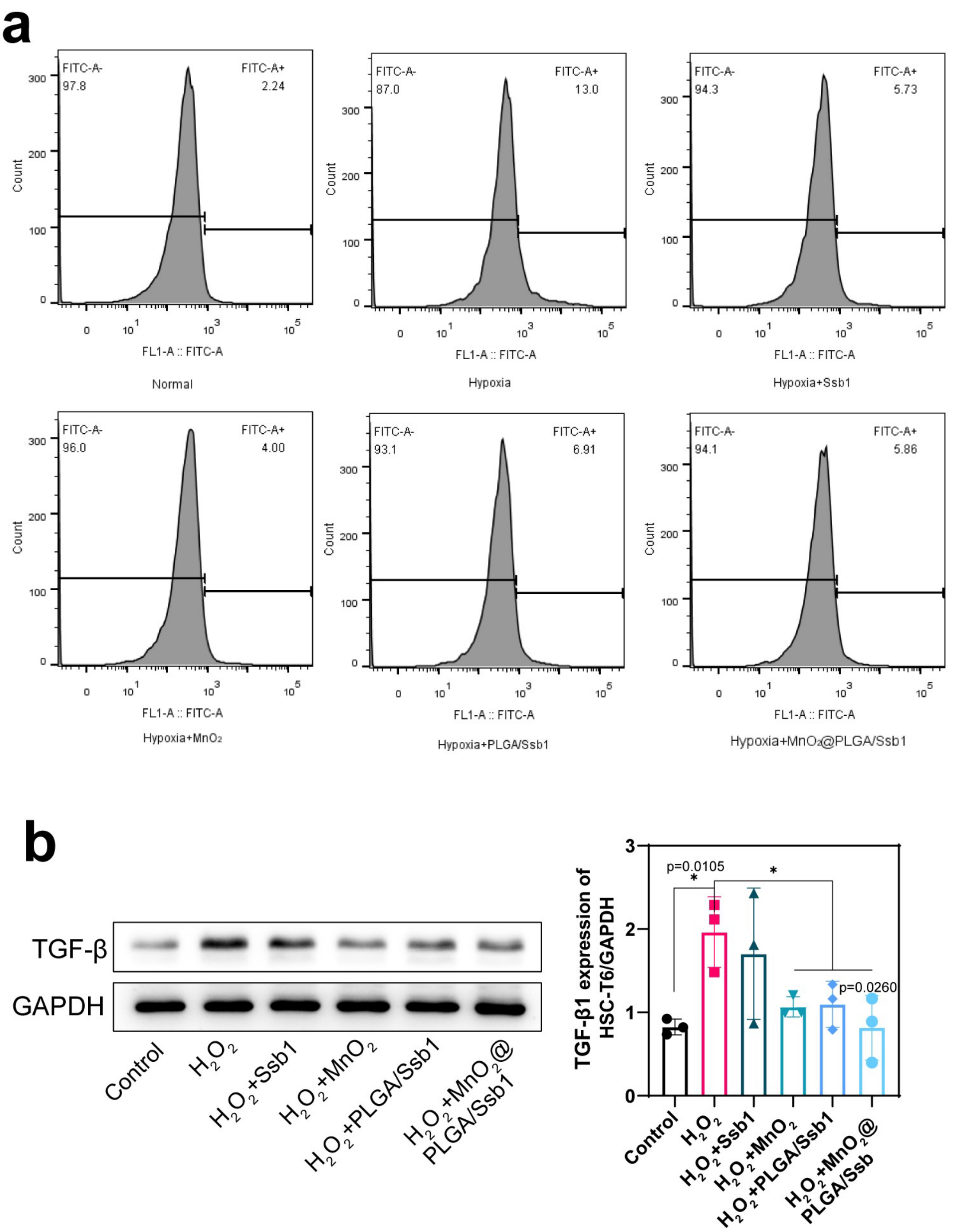

Supplementary Fig. 5. (a) Intracellular ROS levels by flow cytometry analysis. (b) TGF-β1 expression of HSC-T6 with different treatment was measured by WB assay ($n = 3$, Mean ± S.D., $^*p < 0.05$ by Student's t test).

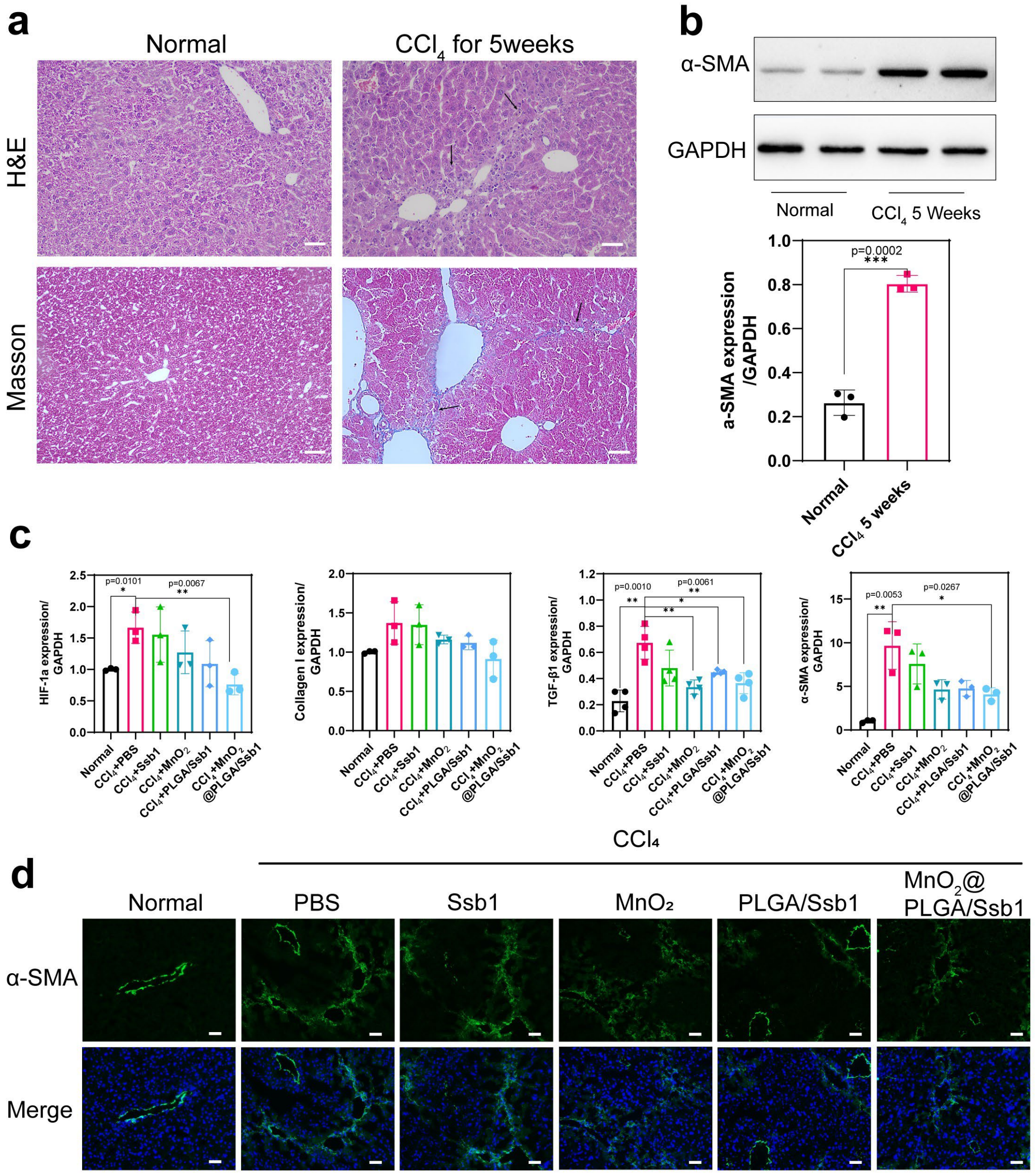

Supplementary Fig. 6. (a) H&E and Masson staining of fibrotic mice with 40 % CCl_4 injection for 5 weeks. Scale bar: 50 μm. (b) Hepatic α-SMA expression of fibrotic mice treated with 40 % CCl_4 for 5 Weeks was measured by western blot assay (n = 3, Mean ± S.D.). (c) Densitometric quantification of data shown in Fig. 6c (n = 3, Mean ± S.D., $*p < 0.05$, $**p < 0.01$; $***p < 0.001$ by Student's t test). (d) Hepatic α-SMA was evaluated by immunofluorescent staining (scale bar, 50 μm).

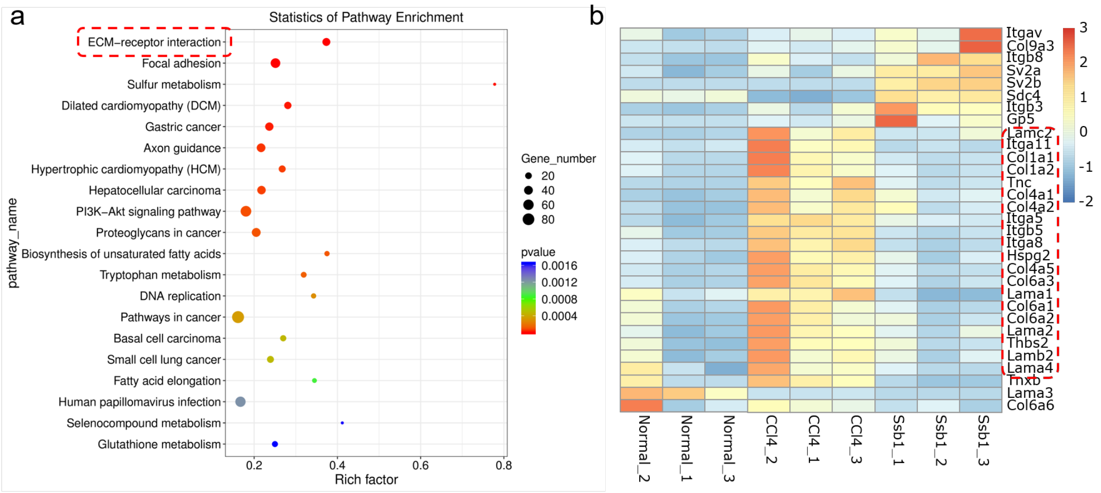

Supplementary Fig. 7. (a) KEGG enrichment of Ssb1 treated mice. (b) Heatmap of differently expressed genes related to “ECM-receptor interaction” pathway (using log base 2).
