# Supplementary material for: Nanodrug rescues liver fibrosis via synergistic therapy with H2O2 depletion and Saikosaponin b1 sustained release
Source: Commun Biol. 2023 Feb 16;6:184. doi: 10.1038/s42003-023-04473-2 (PMC9935535; doi:10.1038/s42003-023-04473-2)
Supplement: Supplementary file 3 — Description of Additional Supplementary Files [file 42003_2023_4473_MOESM3_ESM.pdf]

Description of Additional Supplementary Files

File name: Supplementary Data 1

Description: Plasmid sequencing results of Nrf2 shRNA.

File name: Supplementary Data 2

Description: Western Blot source data (uncropped blots).

File name: Supplementary Data 3

Description: Source data behind all graphs from main and supplementary figures of the paper.
