# Supplementary figures and images for: Nanodrug rescues liver fibrosis via synergistic therapy with H2O2 depletion and Saikosaponin b1 sustained release
Source: Commun Biol. 2023 Feb 16;6:184. doi: 10.1038/s42003-023-04473-2 (PMC9935535; doi:10.1038/s42003-023-04473-2)

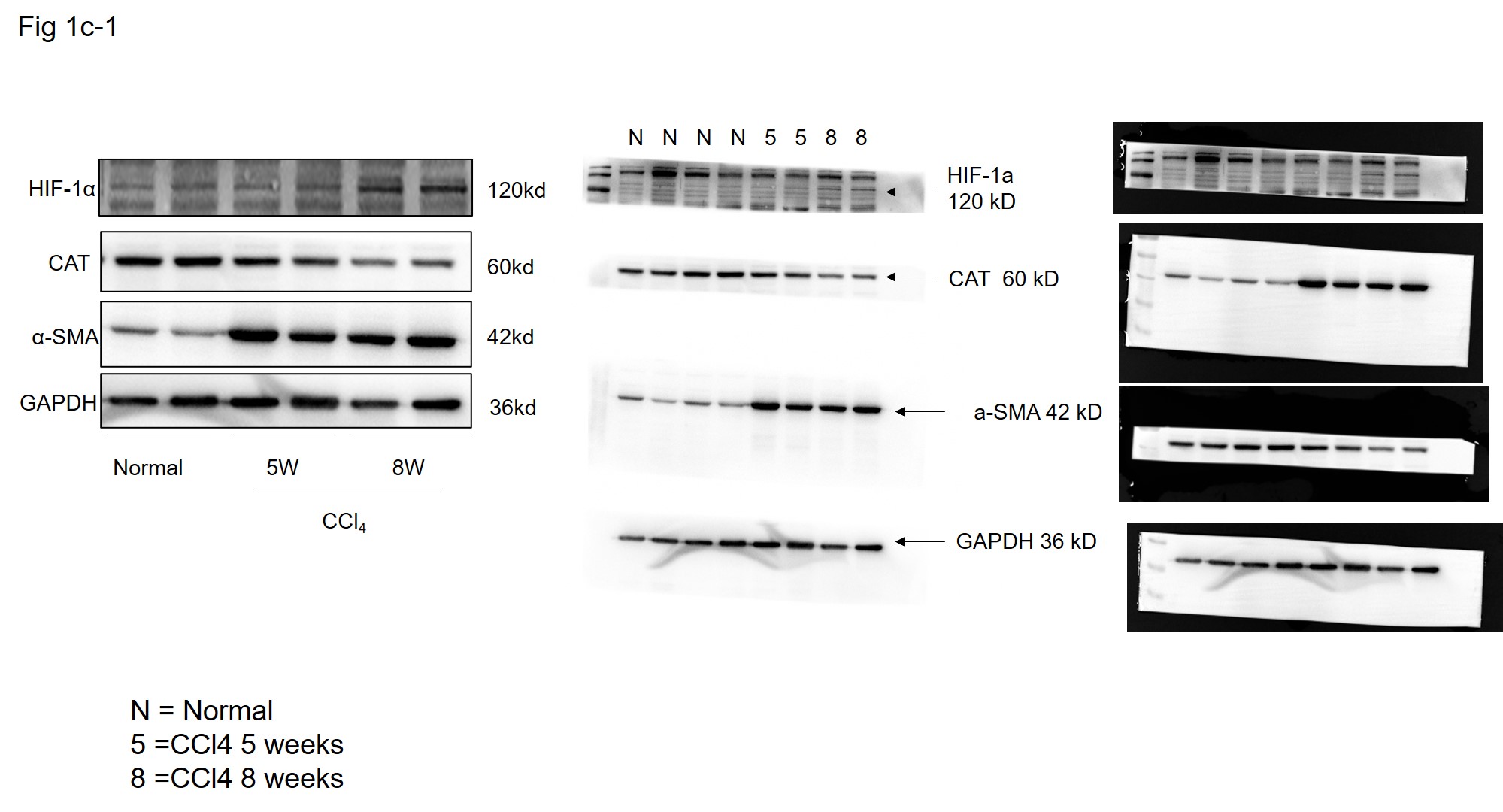

Supplement: Supplementary file 5 — Supplementary Data 2 [file 42003_2023_4473_MOESM5_ESM.zip › Supplementary Data 2/Fig. 1c-1.jpg]

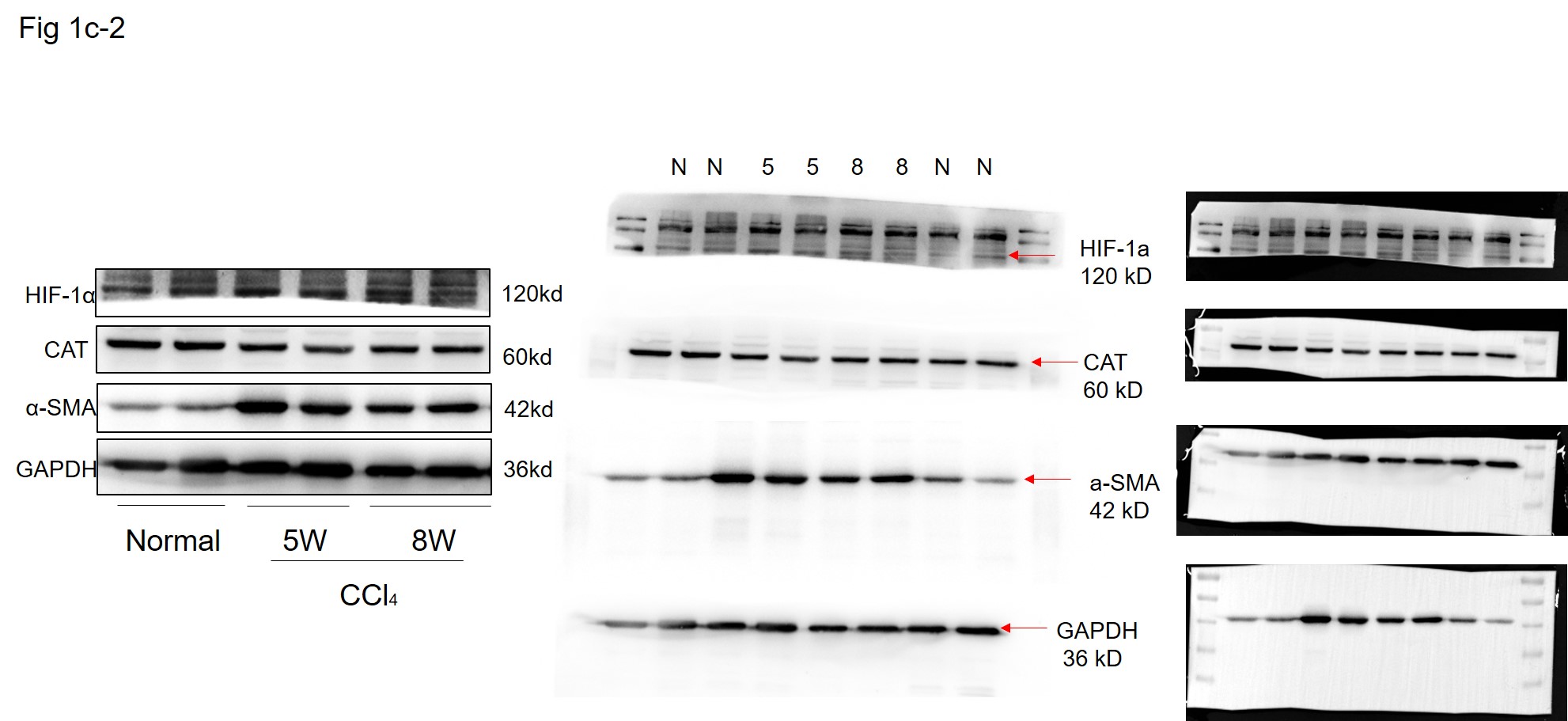

Supplement: Supplementary file 5 — Supplementary Data 2 [file 42003_2023_4473_MOESM5_ESM.zip › Supplementary Data 2/Fig. 1c-2.jpg]

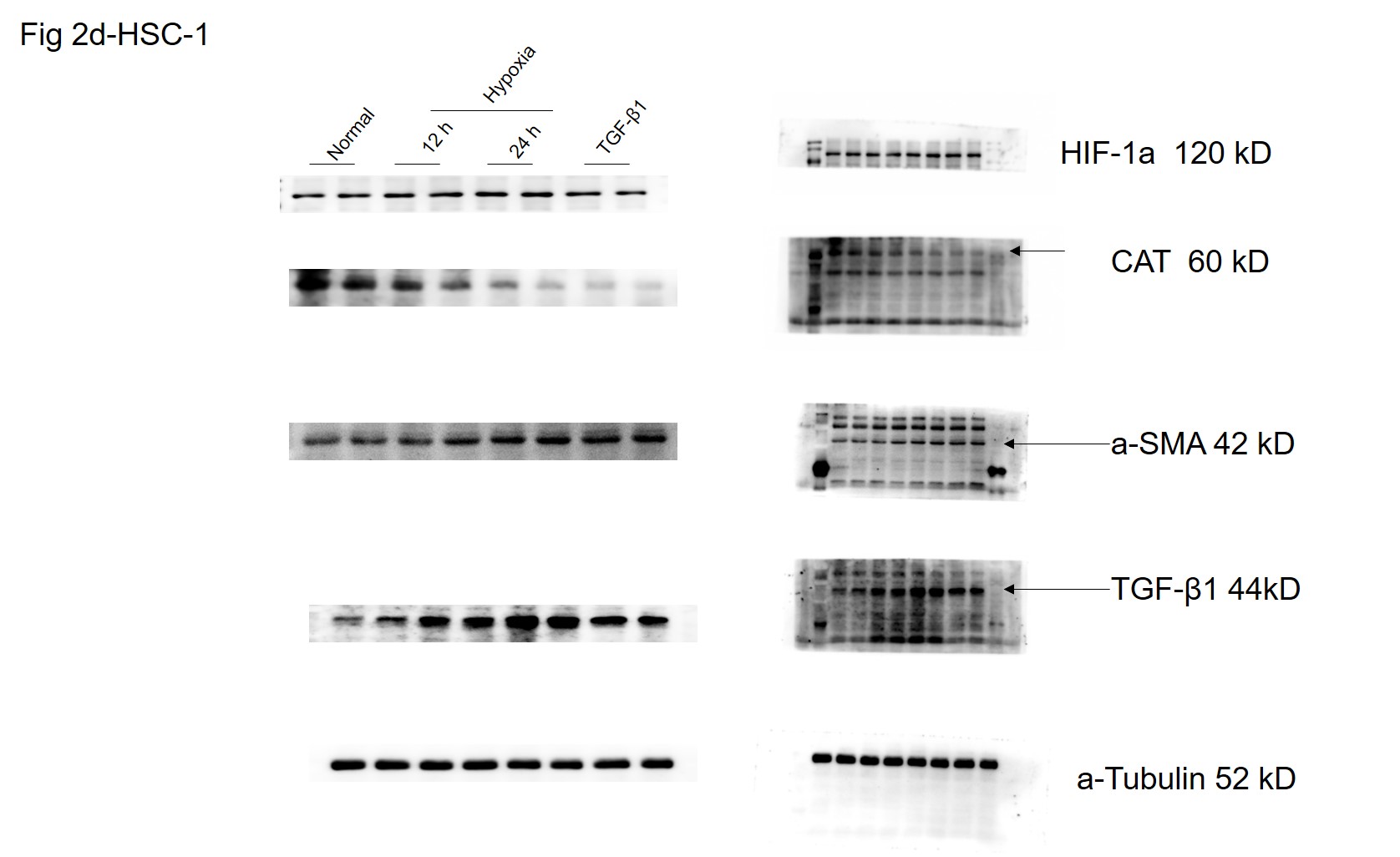

Supplement: Supplementary file 5 — Supplementary Data 2 [file 42003_2023_4473_MOESM5_ESM.zip › Supplementary Data 2/Fig. 2d HSC-T6-1.jpg]

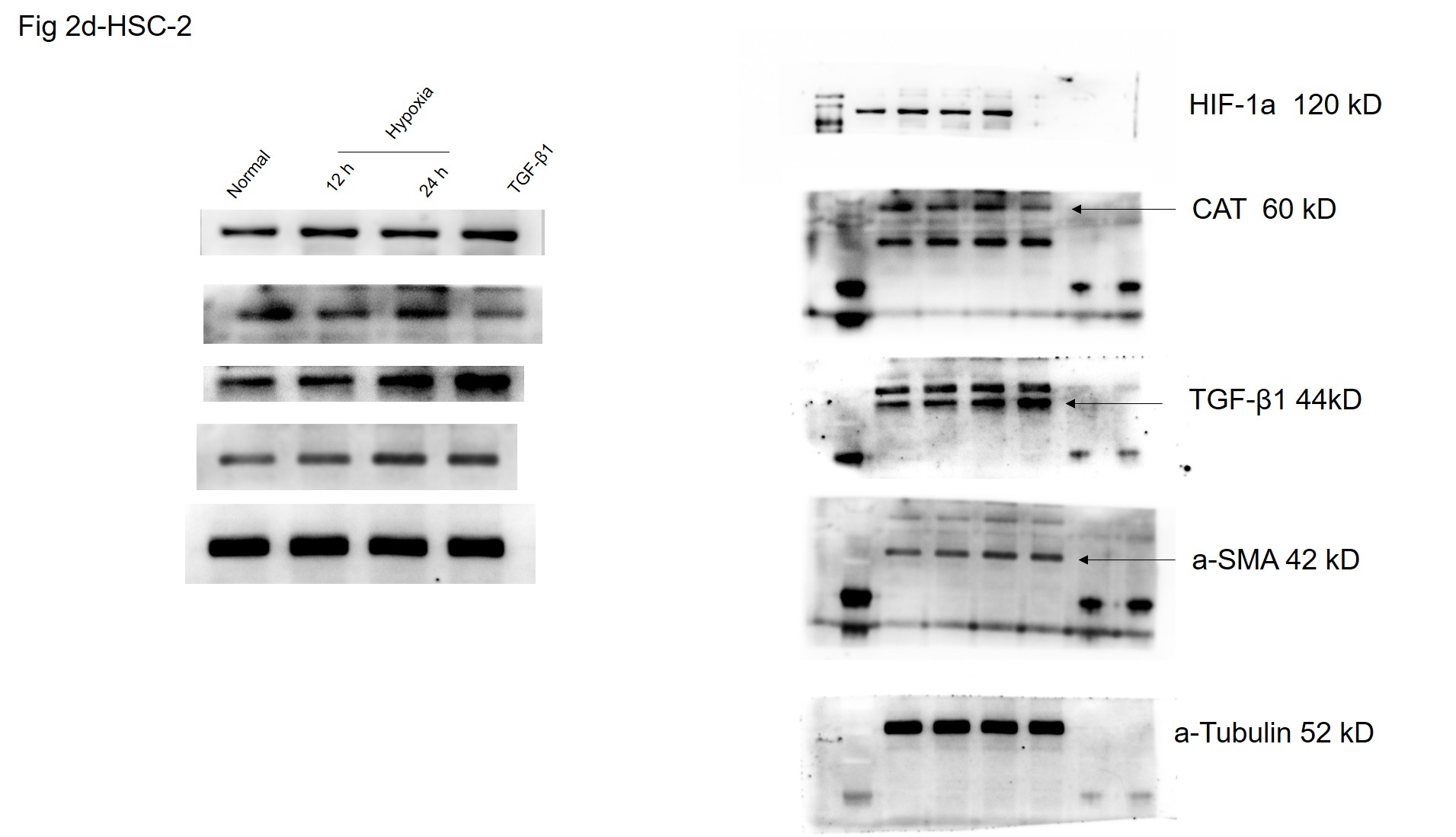

Supplement: Supplementary file 5 — Supplementary Data 2 [file 42003_2023_4473_MOESM5_ESM.zip › Supplementary Data 2/Fig. 2d HSC-T6-2.jpg]

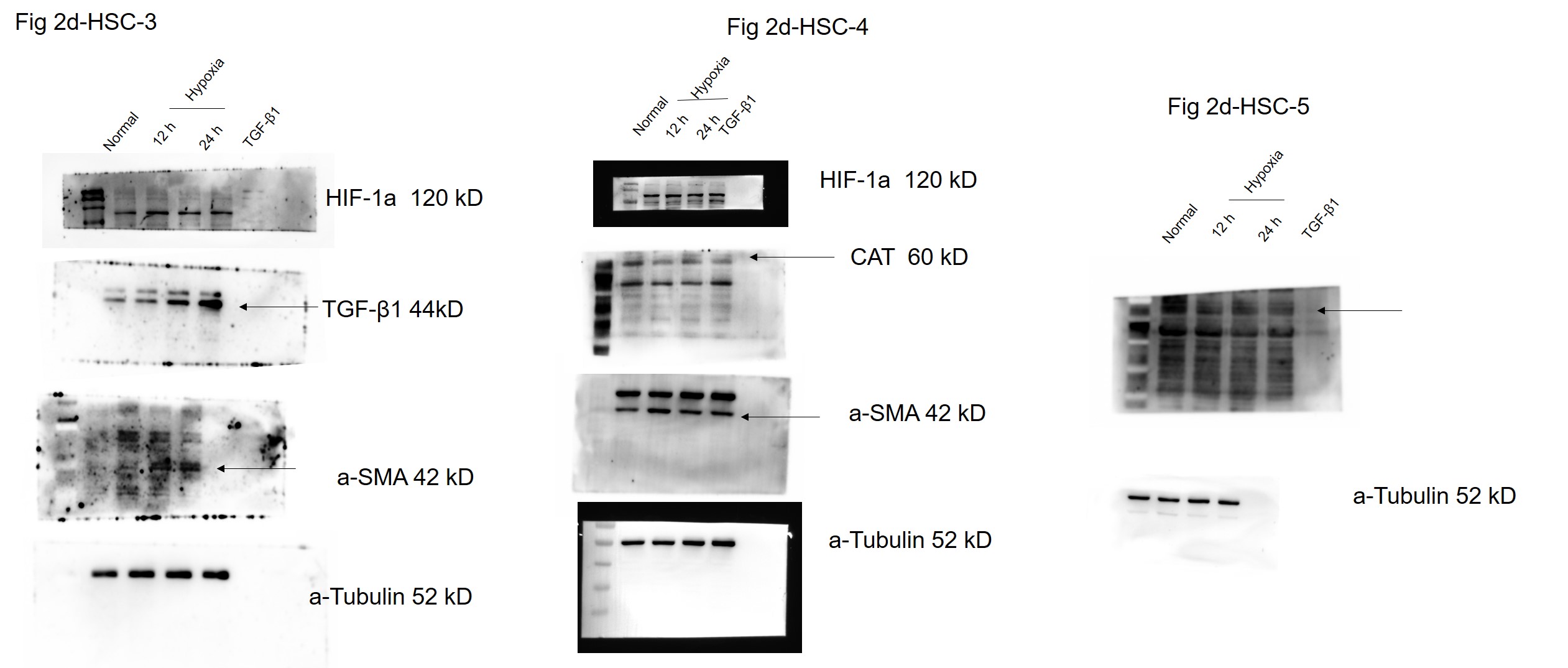

Supplement: Supplementary file 5 — Supplementary Data 2 [file 42003_2023_4473_MOESM5_ESM.zip › Supplementary Data 2/Fig. 2d HSC-T6-3.jpg]

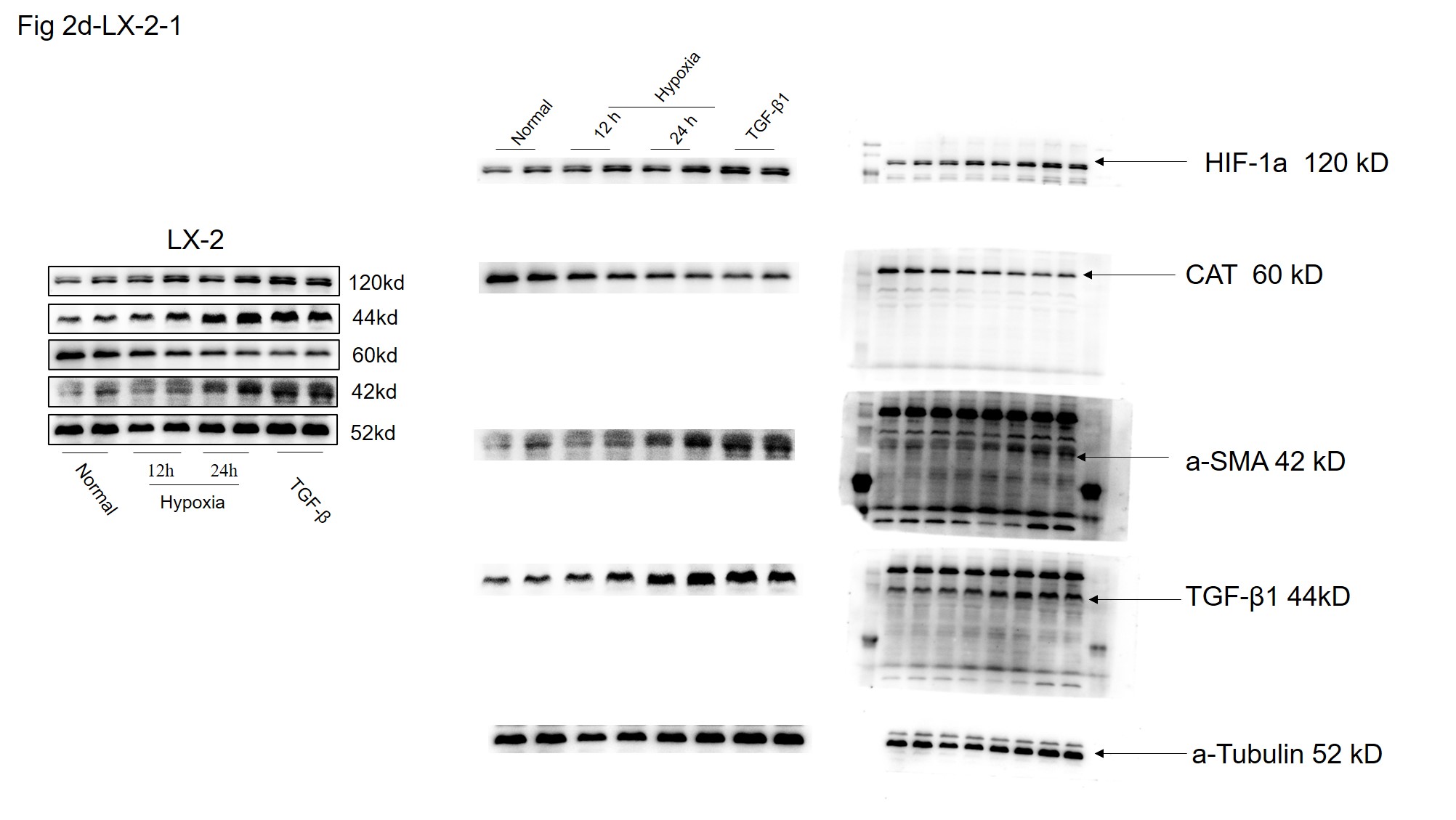

Supplement: Supplementary file 5 — Supplementary Data 2 [file 42003_2023_4473_MOESM5_ESM.zip › Supplementary Data 2/Fig. 2d LX-2-1.jpg]

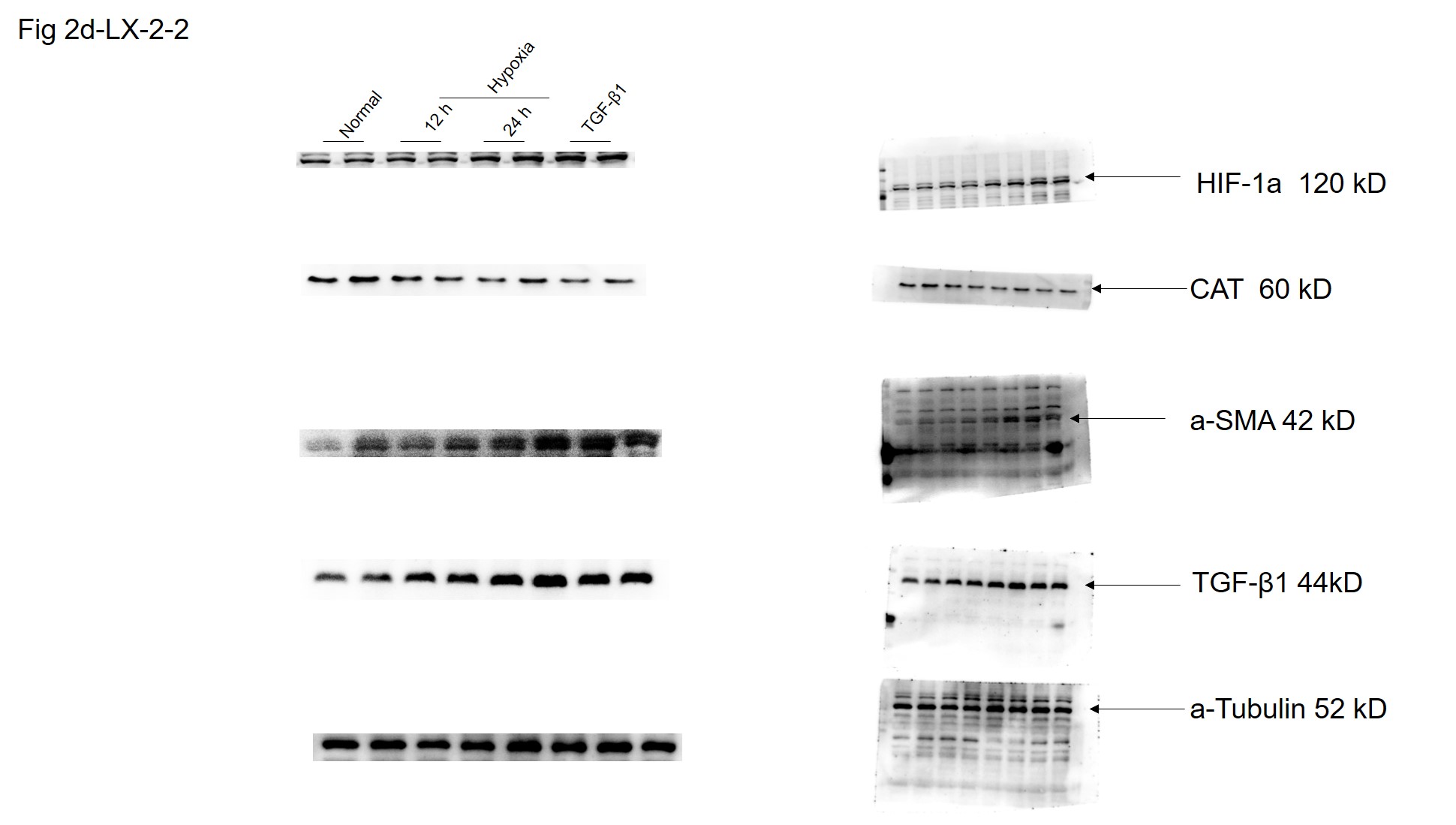

Supplement: Supplementary file 5 — Supplementary Data 2 [file 42003_2023_4473_MOESM5_ESM.zip › Supplementary Data 2/Fig. 2d LX-2-2.jpg]

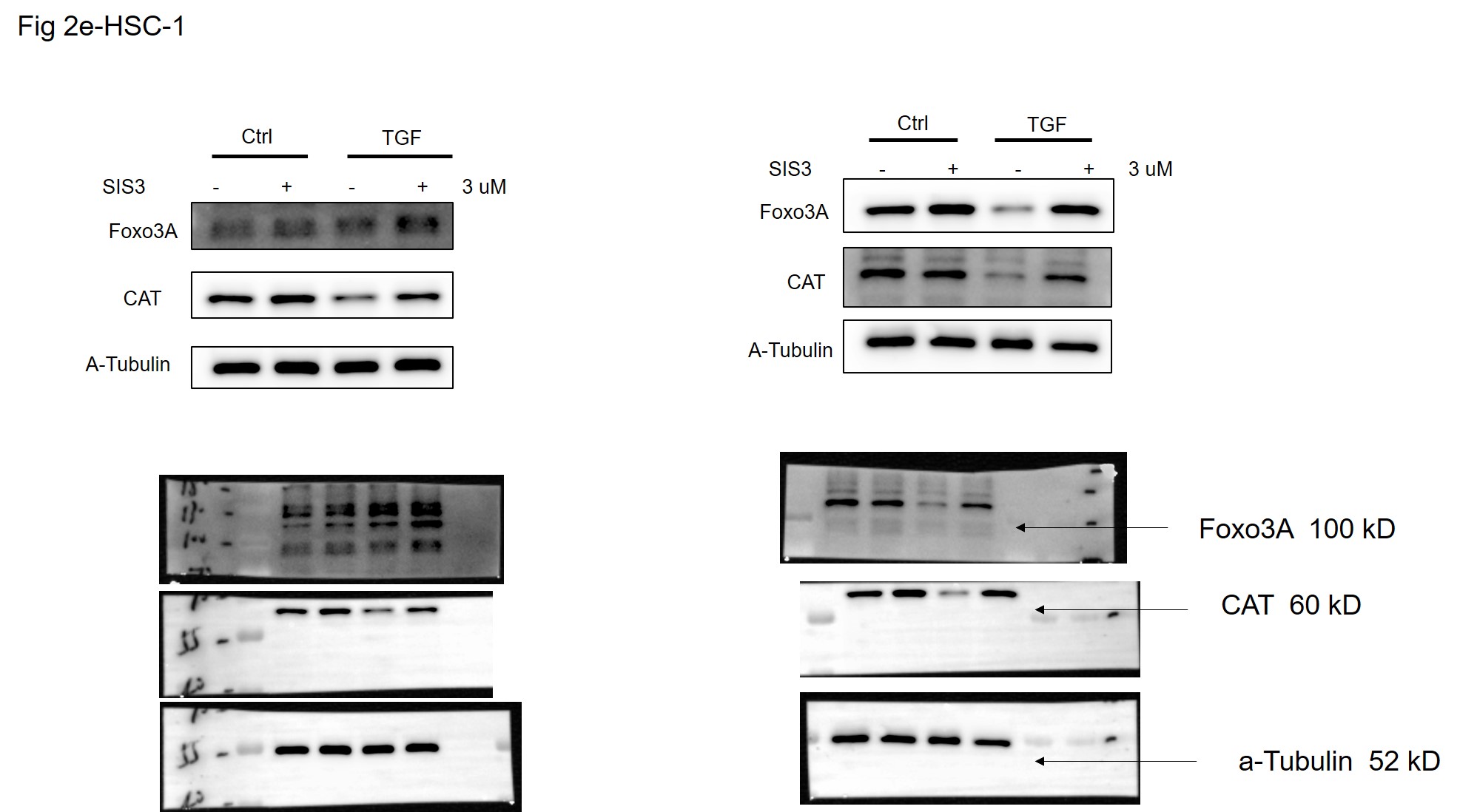

Supplement: Supplementary file 5 — Supplementary Data 2 [file 42003_2023_4473_MOESM5_ESM.zip › Supplementary Data 2/Fig. 2e HSC-T6-1.jpg]

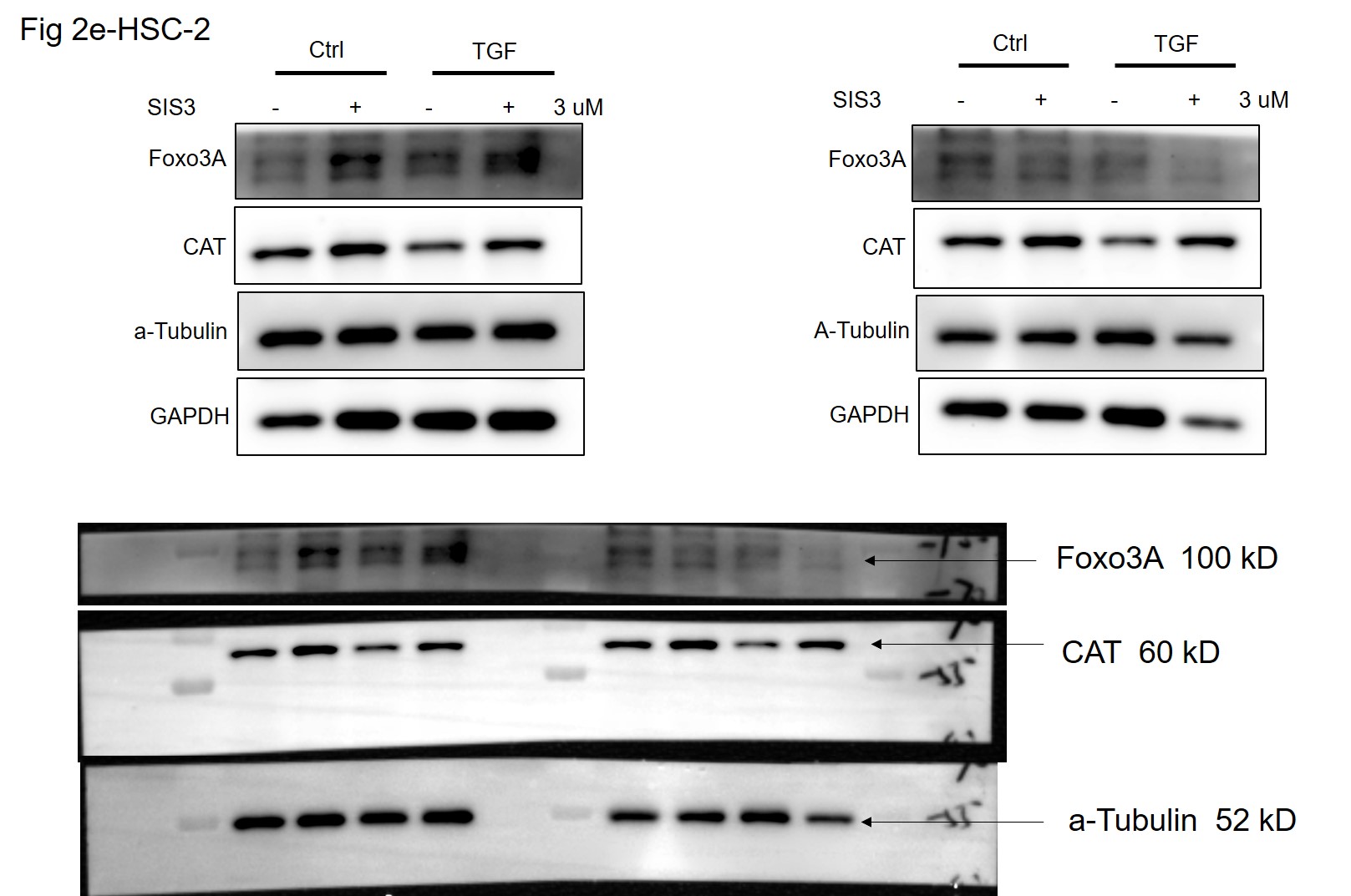

Supplement: Supplementary file 5 — Supplementary Data 2 [file 42003_2023_4473_MOESM5_ESM.zip › Supplementary Data 2/Fig. 2e HSC-T6-2.jpg]

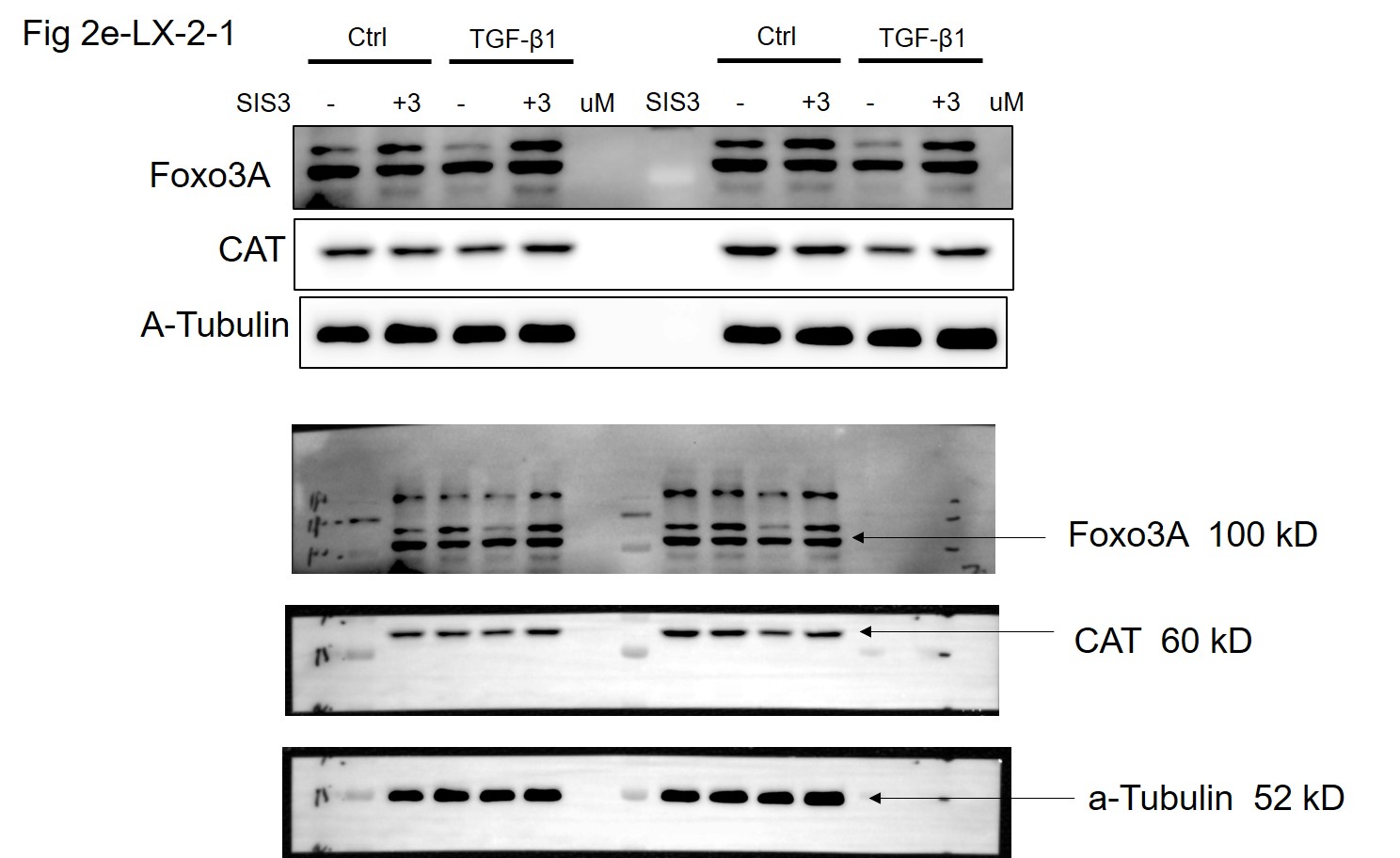

Supplement: Supplementary file 5 — Supplementary Data 2 [file 42003_2023_4473_MOESM5_ESM.zip › Supplementary Data 2/Fig. 2e LX-2-1.jpg]

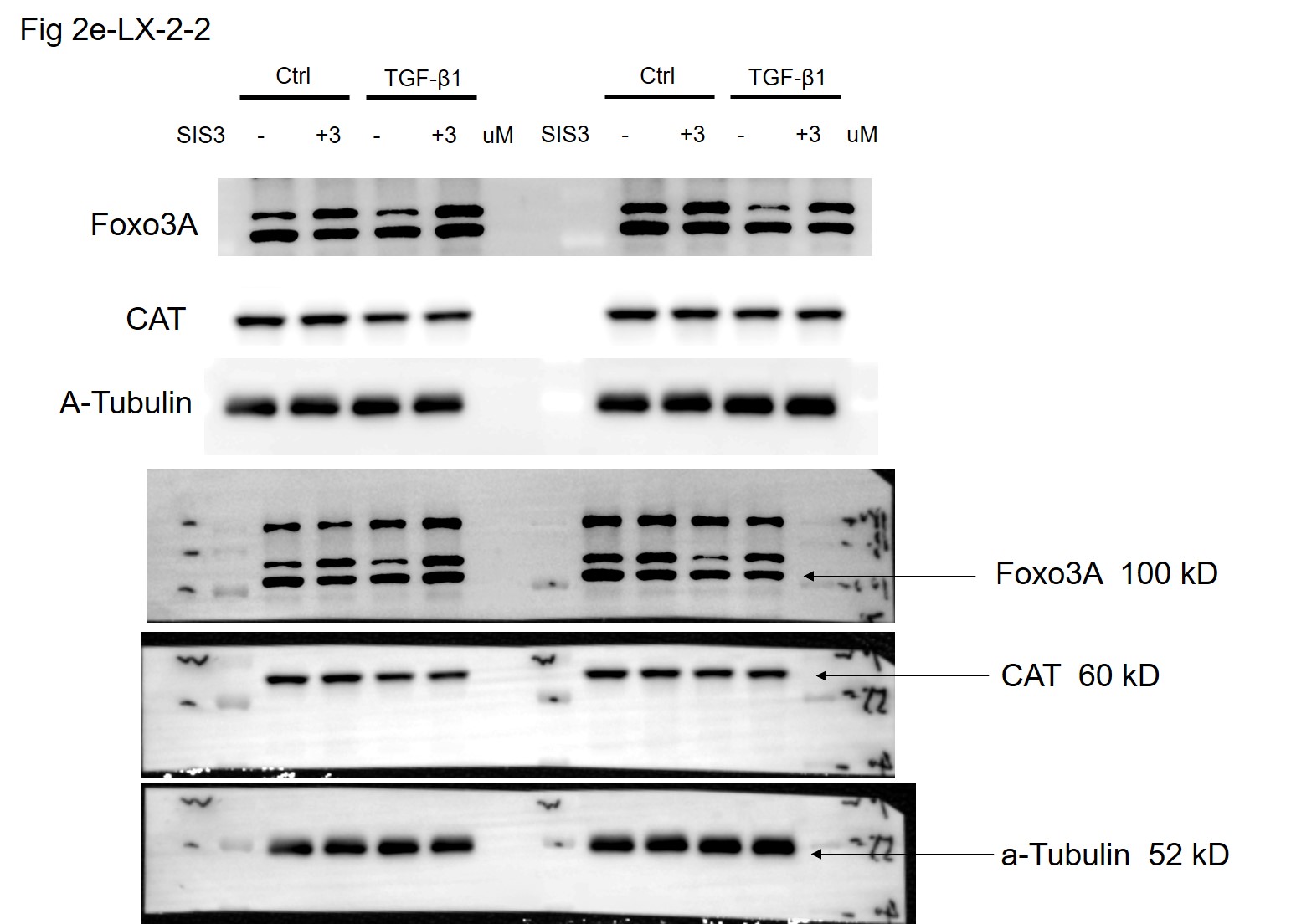

Supplement: Supplementary file 5 — Supplementary Data 2 [file 42003_2023_4473_MOESM5_ESM.zip › Supplementary Data 2/Fig. 2e LX-2-2.jpg]

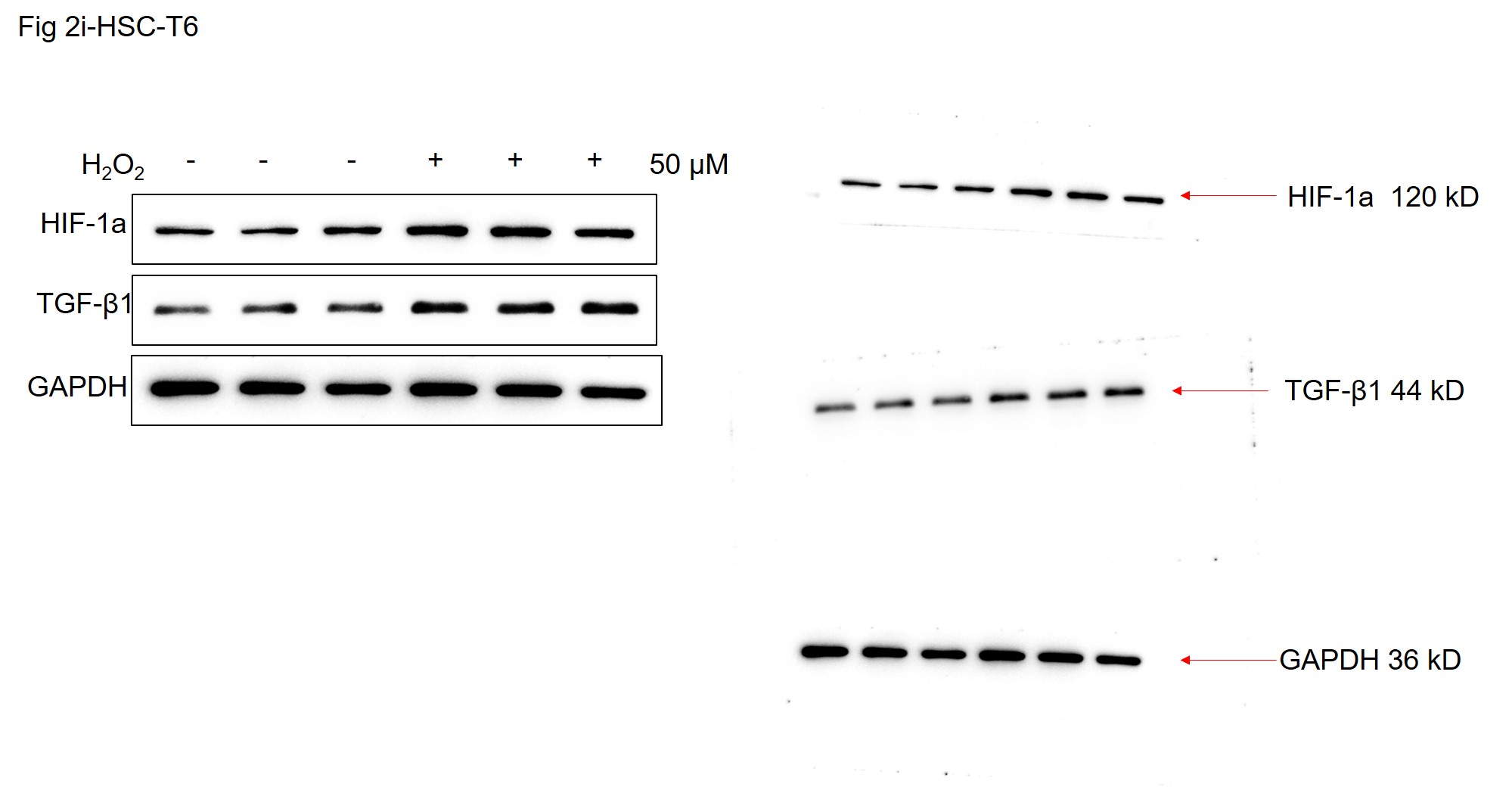

Supplement: Supplementary file 5 — Supplementary Data 2 [file 42003_2023_4473_MOESM5_ESM.zip › Supplementary Data 2/Fig. 2i HSC-T6.jpg]

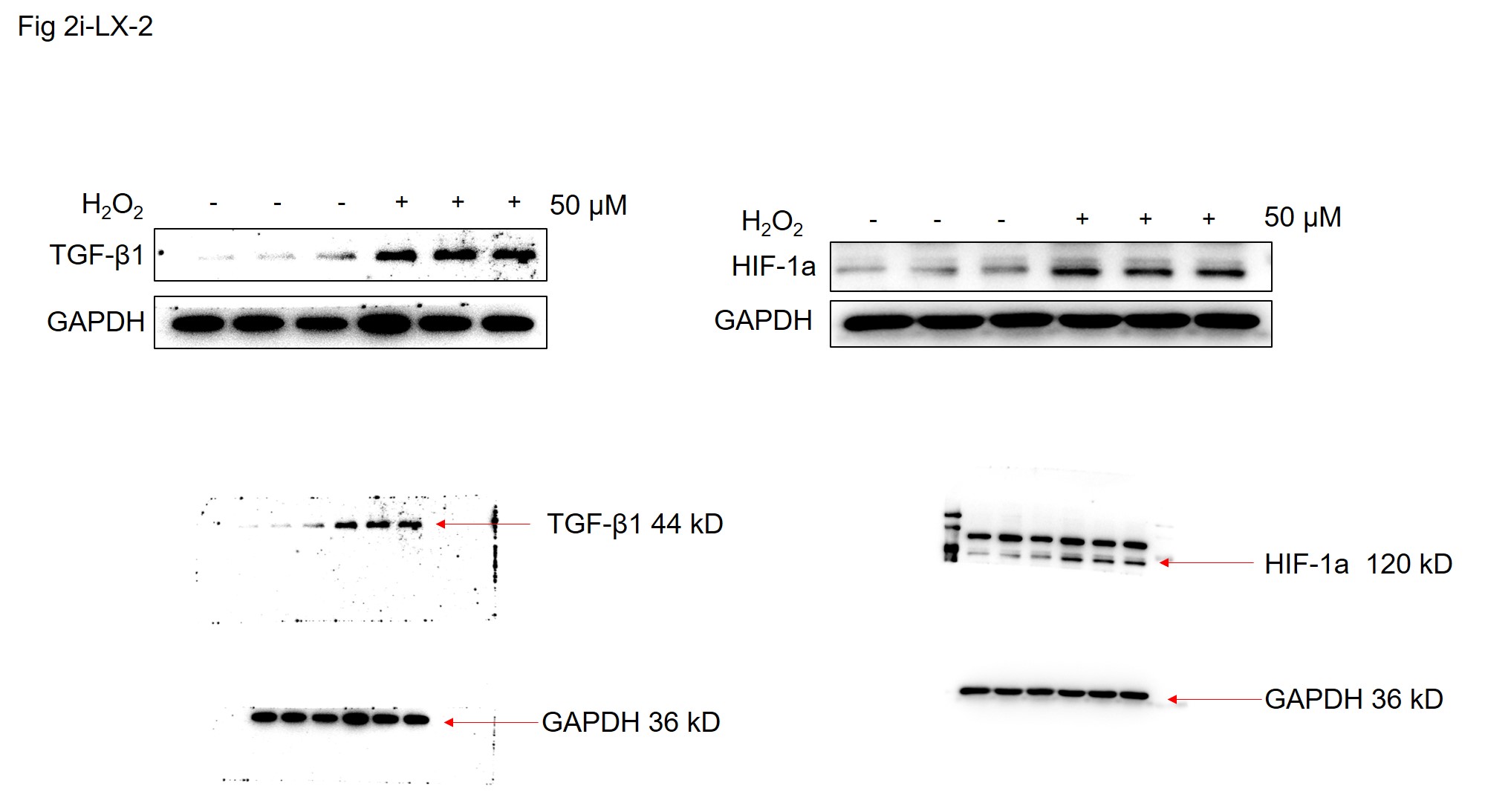

Supplement: Supplementary file 5 — Supplementary Data 2 [file 42003_2023_4473_MOESM5_ESM.zip › Supplementary Data 2/Fig. 2i LX-2.jpg]

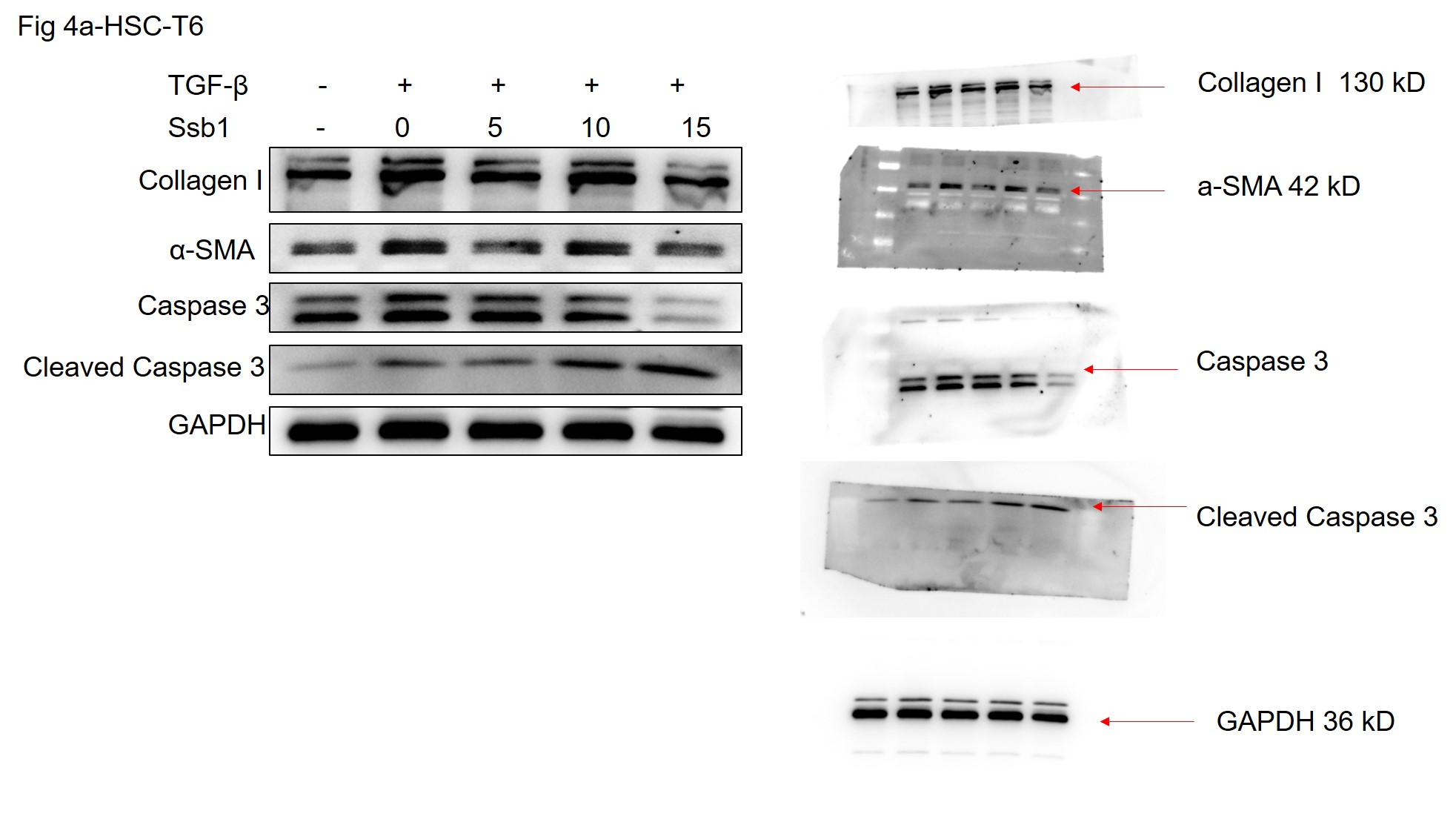

Supplement: Supplementary file 5 — Supplementary Data 2 [file 42003_2023_4473_MOESM5_ESM.zip › Supplementary Data 2/Fig. 4a HSC-T6.jpg]

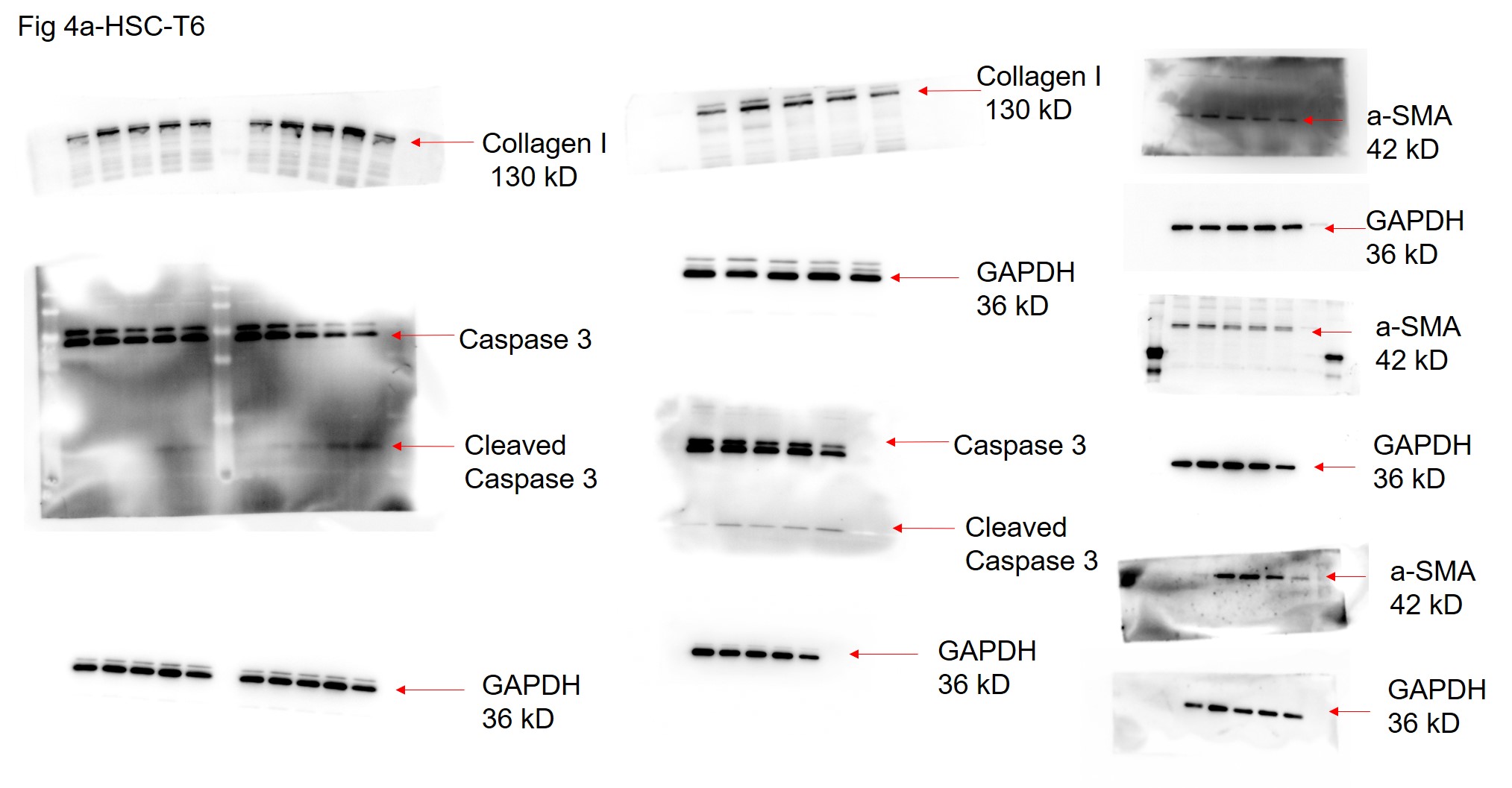

Supplement: Supplementary file 5 — Supplementary Data 2 [file 42003_2023_4473_MOESM5_ESM.zip › Supplementary Data 2/Fig. 4a HSC-T6-1.jpg]

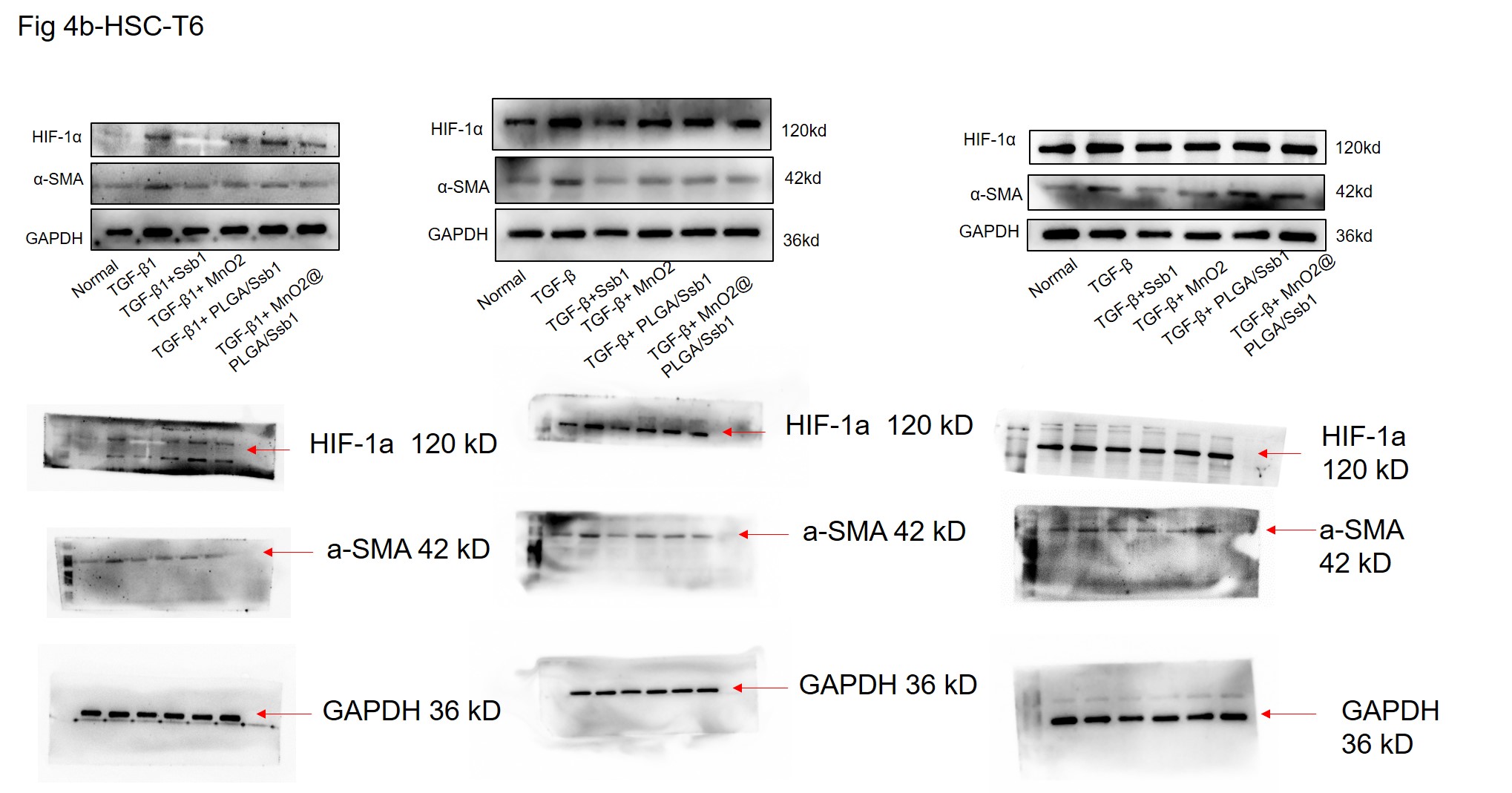

Supplement: Supplementary file 5 — Supplementary Data 2 [file 42003_2023_4473_MOESM5_ESM.zip › Supplementary Data 2/Fig. 4b HSC-T6.jpg]

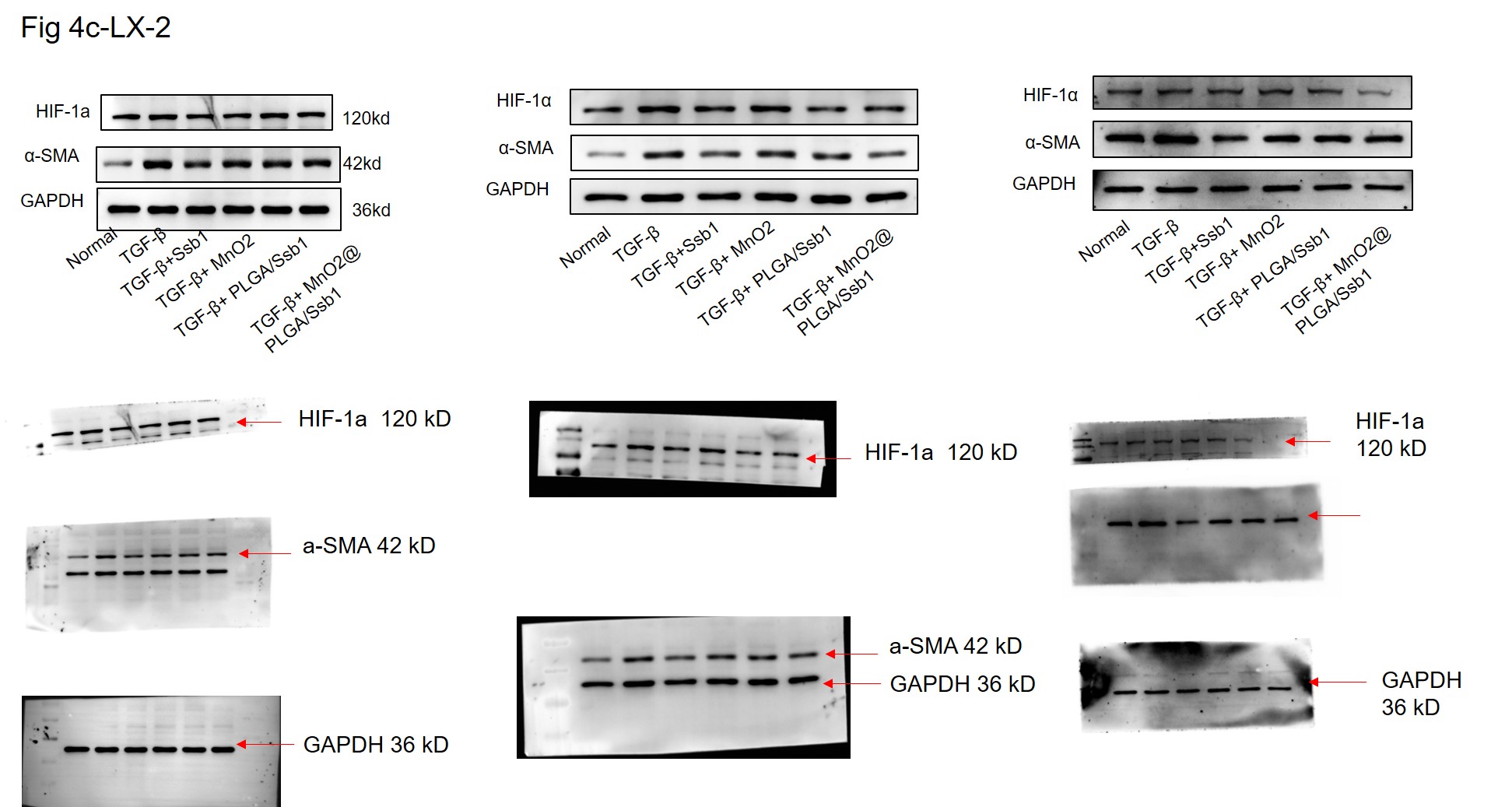

Supplement: Supplementary file 5 — Supplementary Data 2 [file 42003_2023_4473_MOESM5_ESM.zip › Supplementary Data 2/Fig. 4c LX-2.jpg]

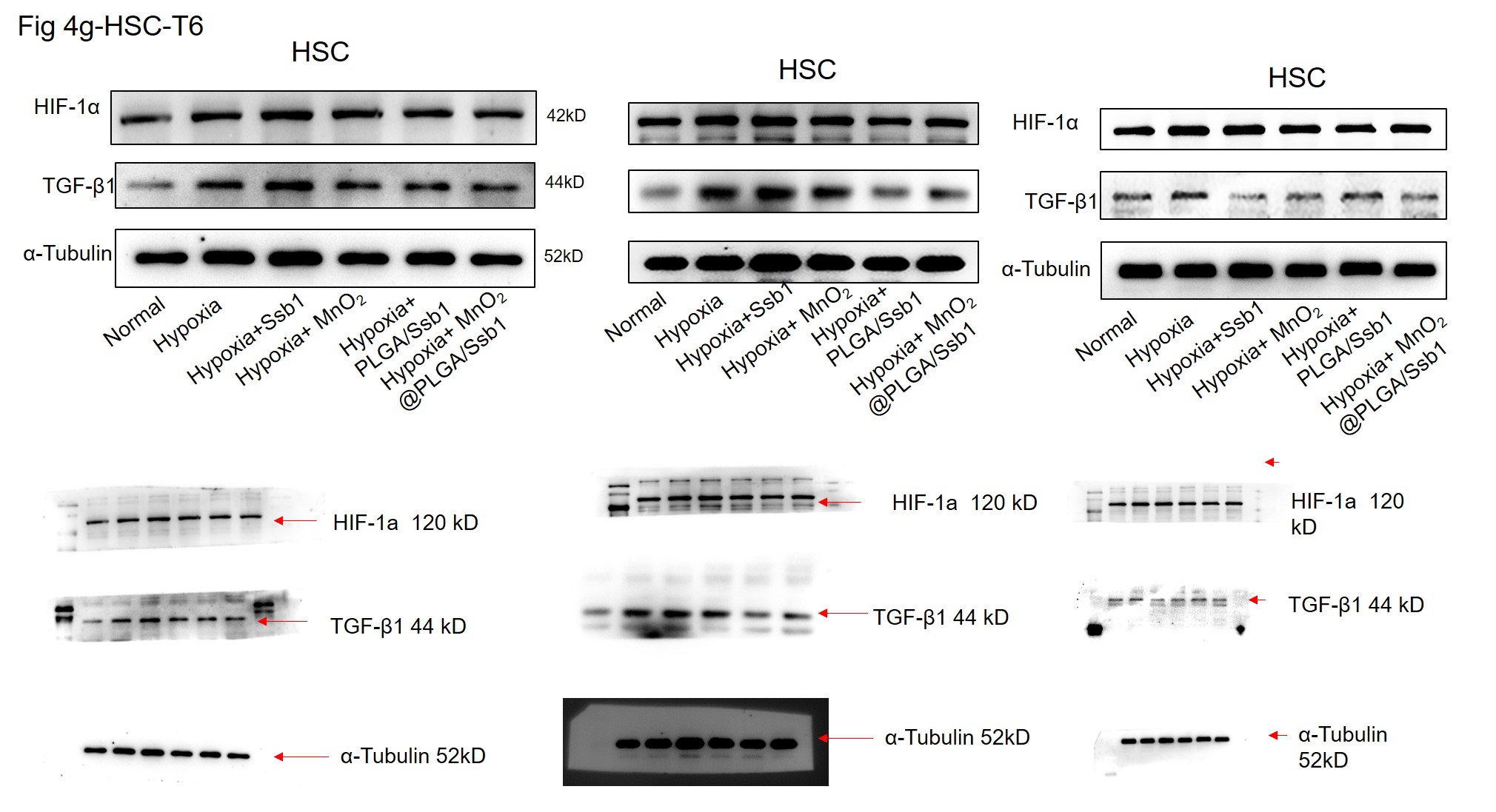

Supplement: Supplementary file 5 — Supplementary Data 2 [file 42003_2023_4473_MOESM5_ESM.zip › Supplementary Data 2/Fig. 4g HSC-T6.jpg]

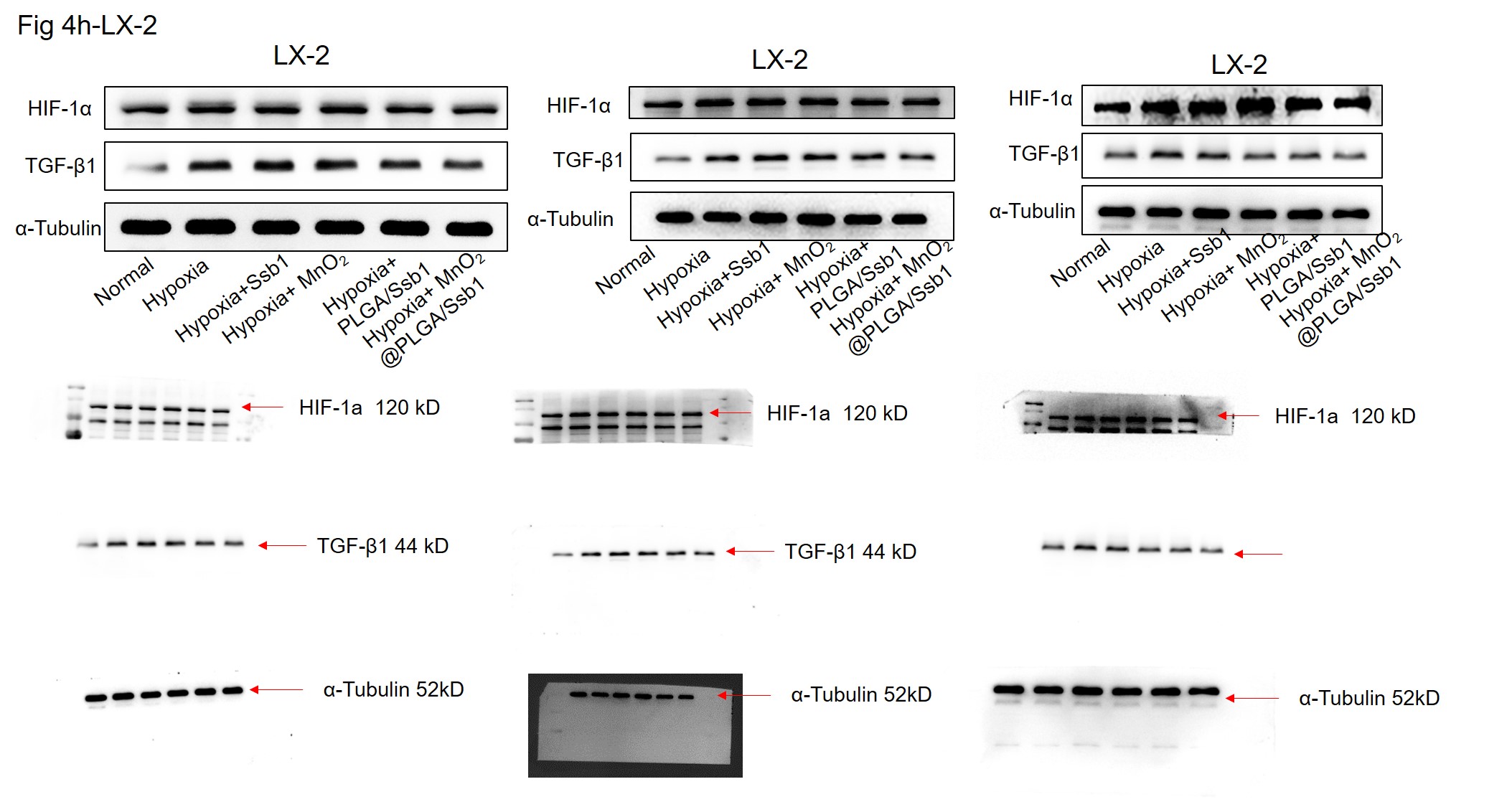

Supplement: Supplementary file 5 — Supplementary Data 2 [file 42003_2023_4473_MOESM5_ESM.zip › Supplementary Data 2/Fig. 4h LX-2.jpg]

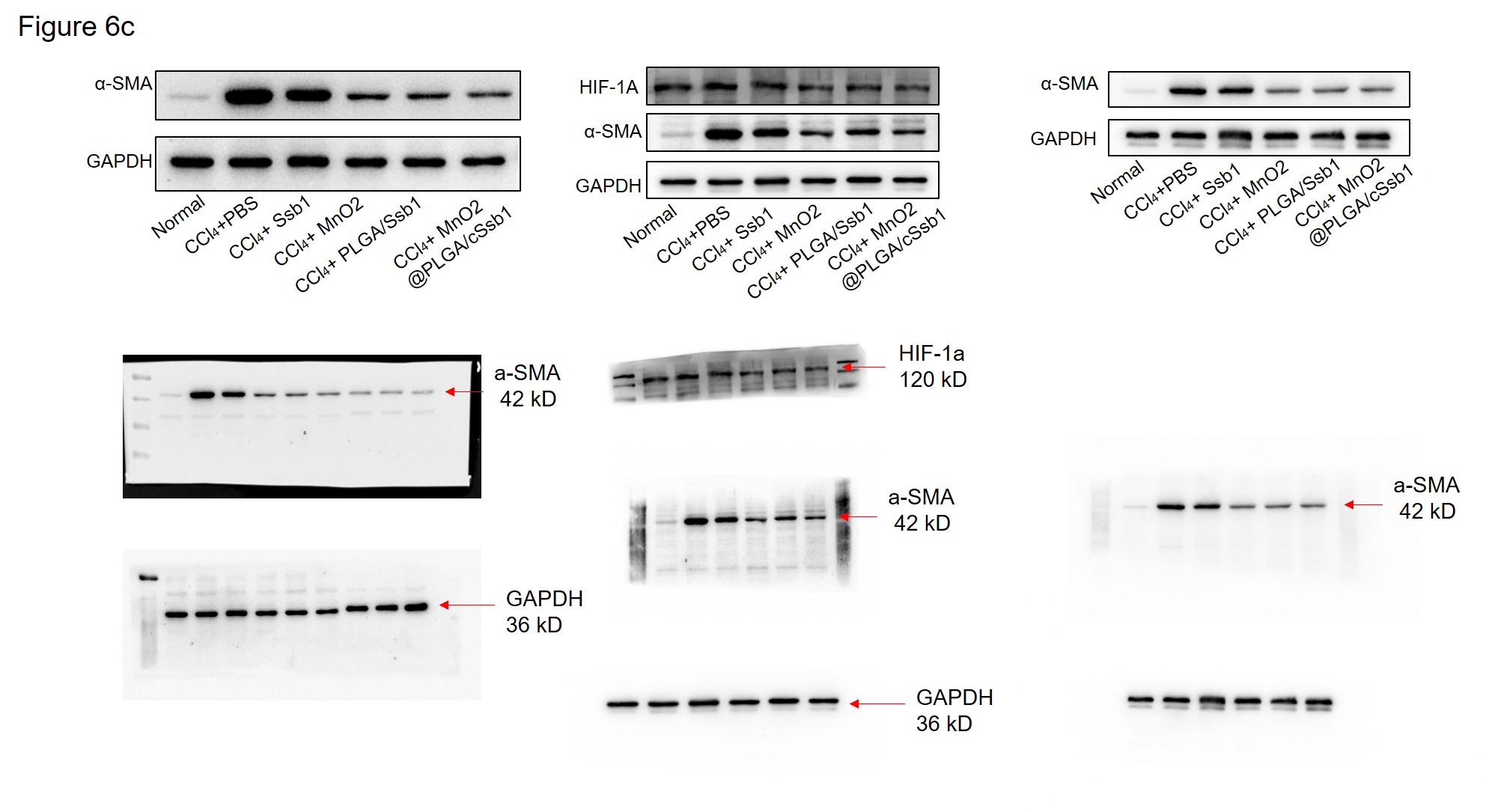

Supplement: Supplementary file 5 — Supplementary Data 2 [file 42003_2023_4473_MOESM5_ESM.zip › Supplementary Data 2/Fig. 6c.jpg]

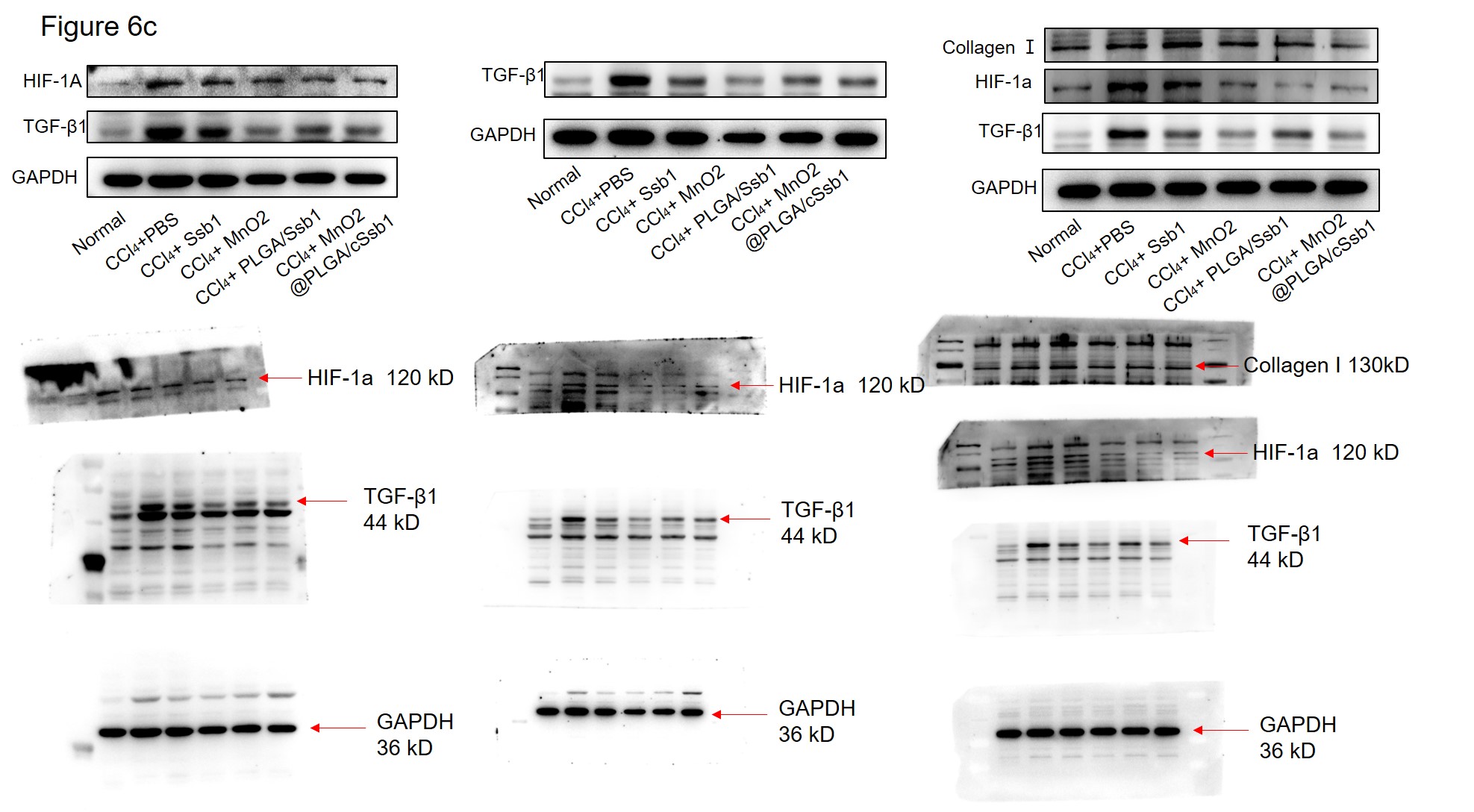

Supplement: Supplementary file 5 — Supplementary Data 2 [file 42003_2023_4473_MOESM5_ESM.zip › Supplementary Data 2/Fig. 6c-1.jpg]

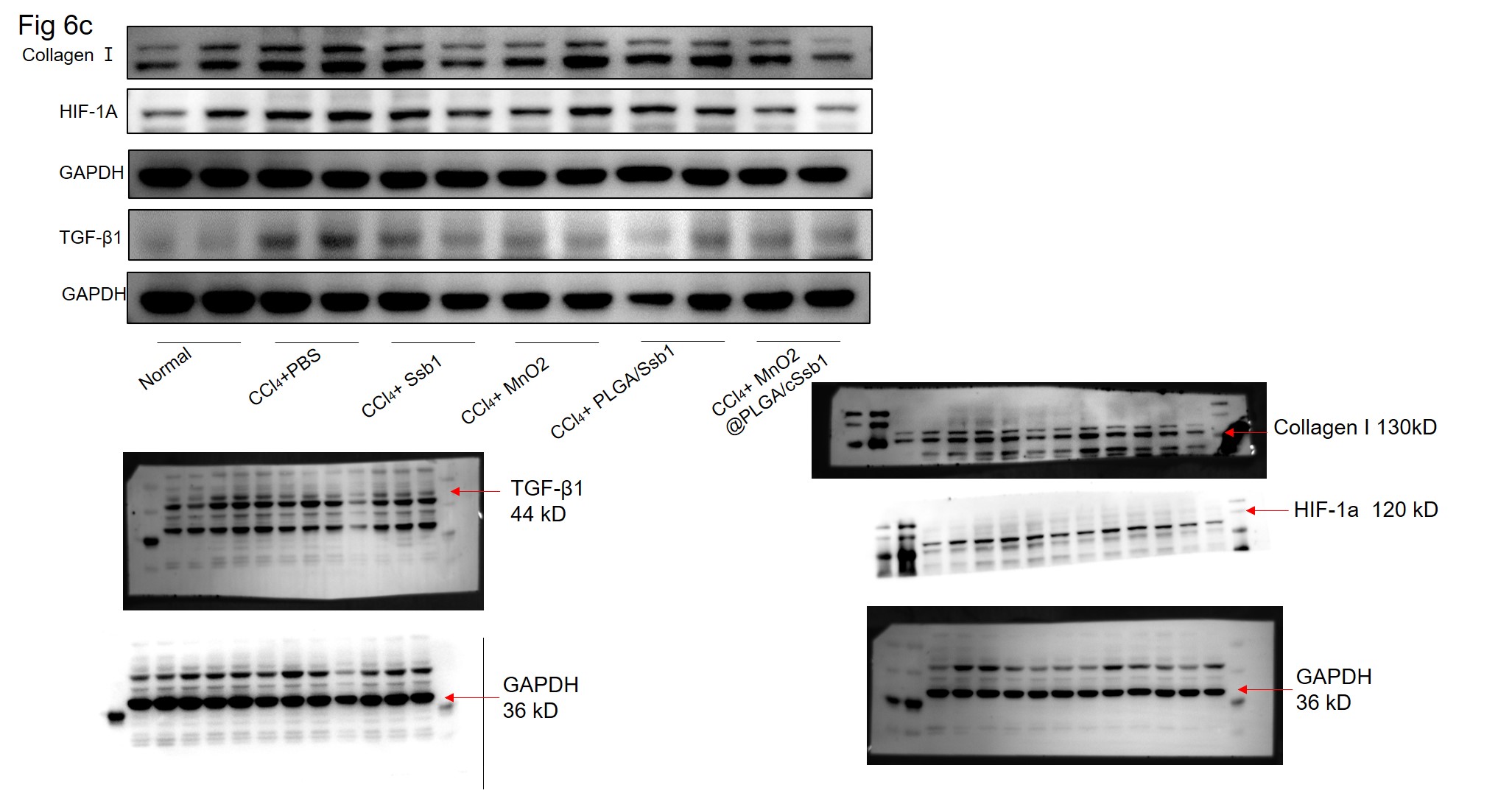

Supplement: Supplementary file 5 — Supplementary Data 2 [file 42003_2023_4473_MOESM5_ESM.zip › Supplementary Data 2/Fig. 6c-2.jpg]

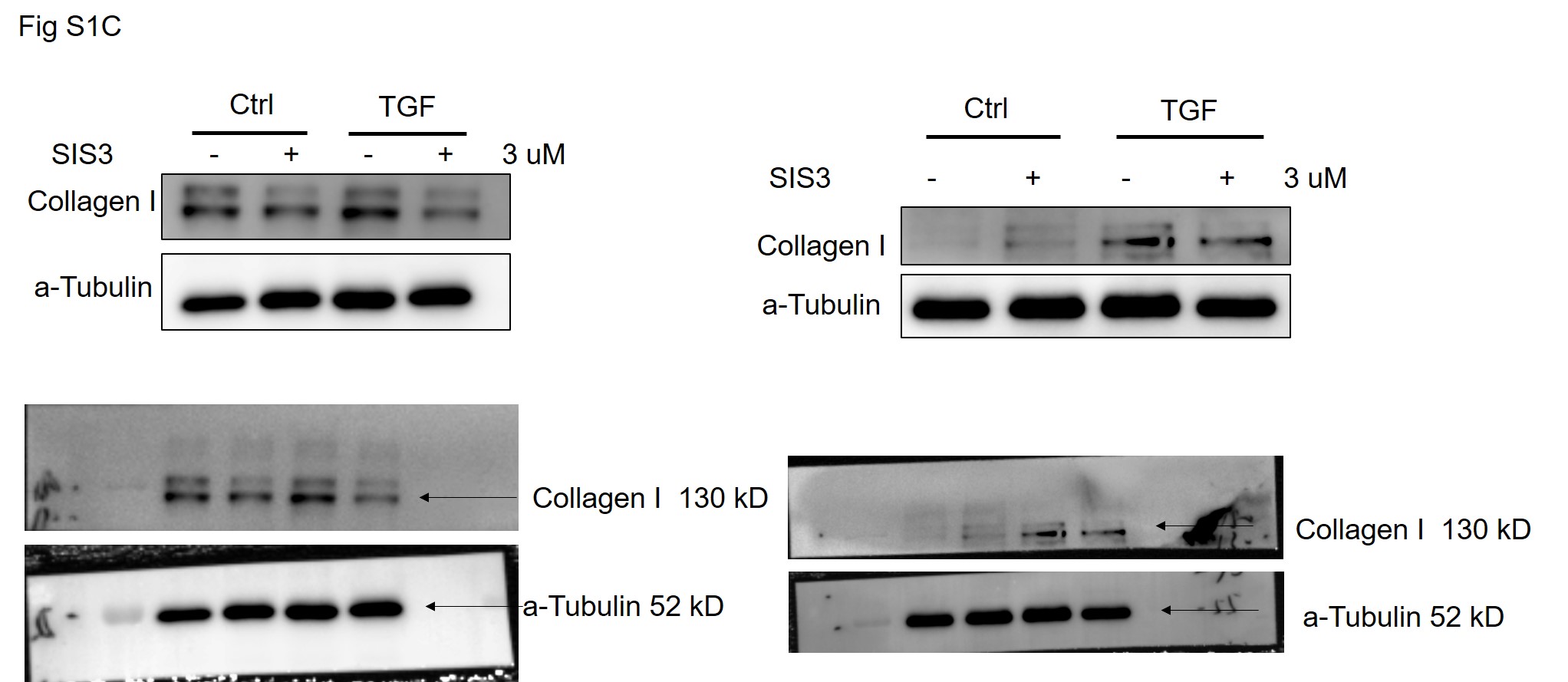

Supplement: Supplementary file 5 — Supplementary Data 2 [file 42003_2023_4473_MOESM5_ESM.zip › Supplementary Data 2/Fig. S1c.jpg]

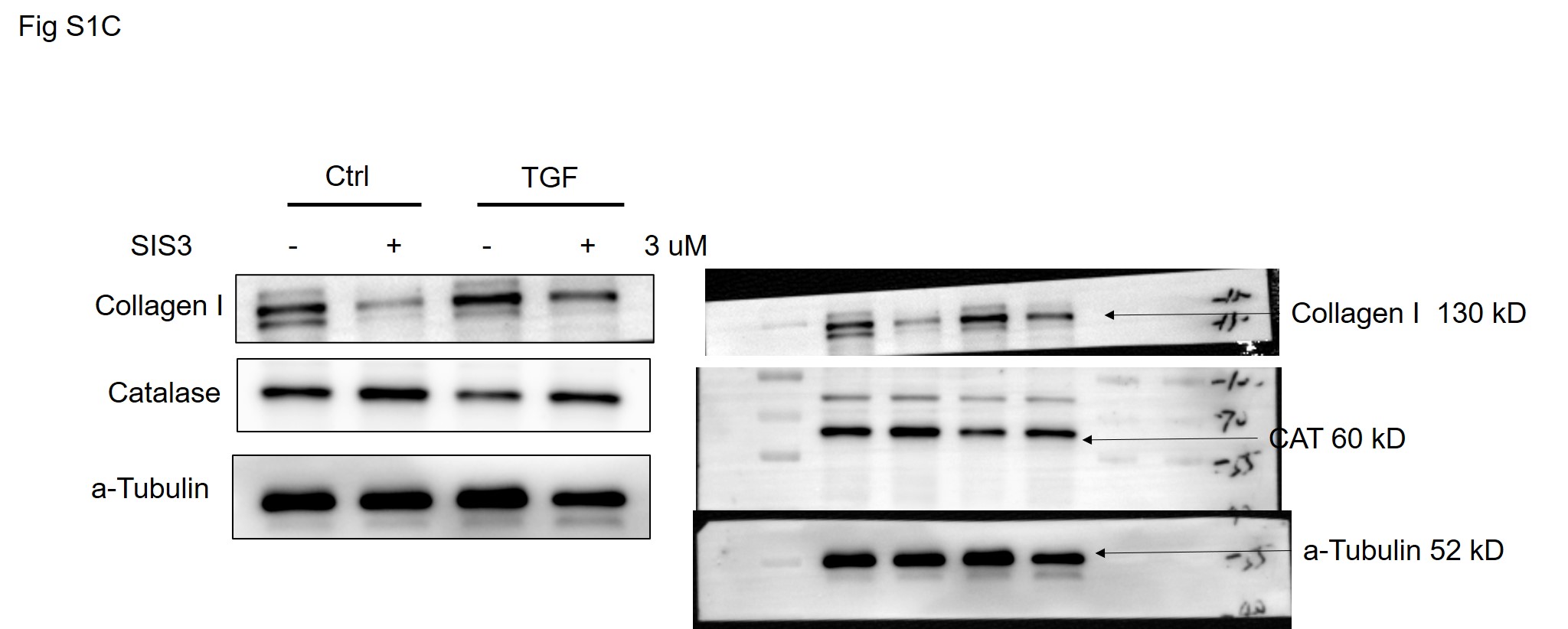

Supplement: Supplementary file 5 — Supplementary Data 2 [file 42003_2023_4473_MOESM5_ESM.zip › Supplementary Data 2/Fig. S1c-1.jpg]

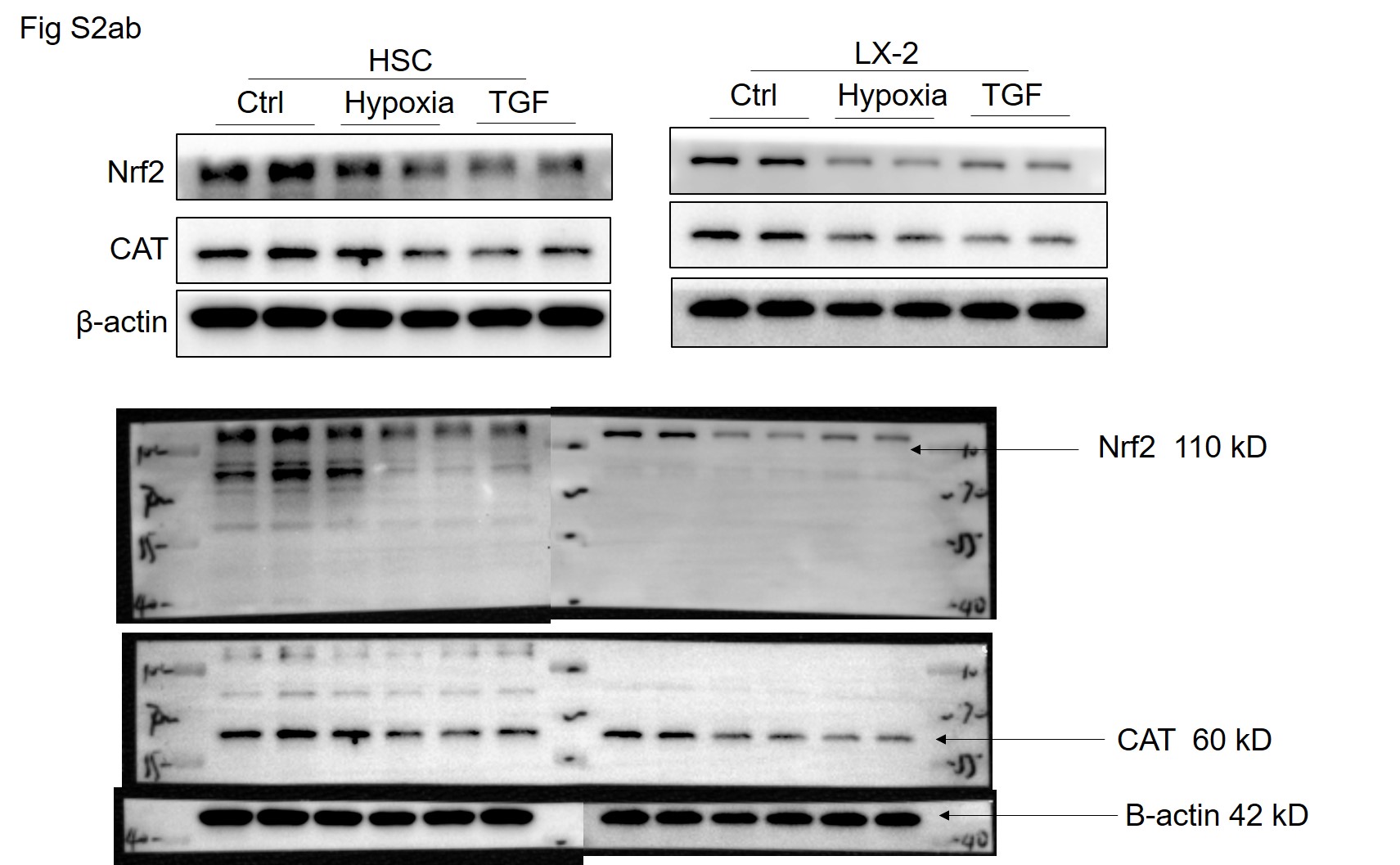

Supplement: Supplementary file 5 — Supplementary Data 2 [file 42003_2023_4473_MOESM5_ESM.zip › Supplementary Data 2/Fig. S2a and S2b.jpg]

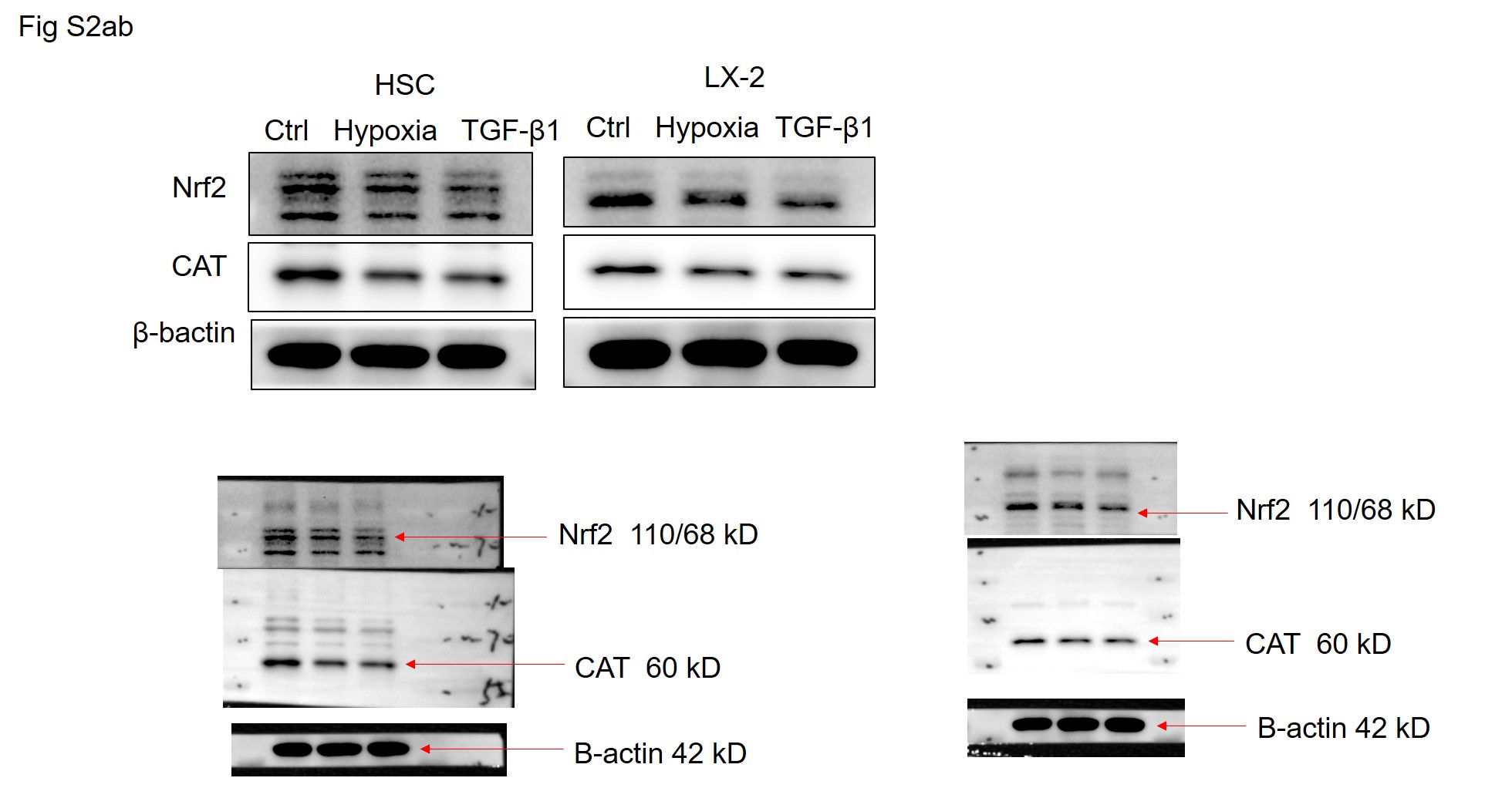

Supplement: Supplementary file 5 — Supplementary Data 2 [file 42003_2023_4473_MOESM5_ESM.zip › Supplementary Data 2/Fig. S2a and S2b-1.jpg]

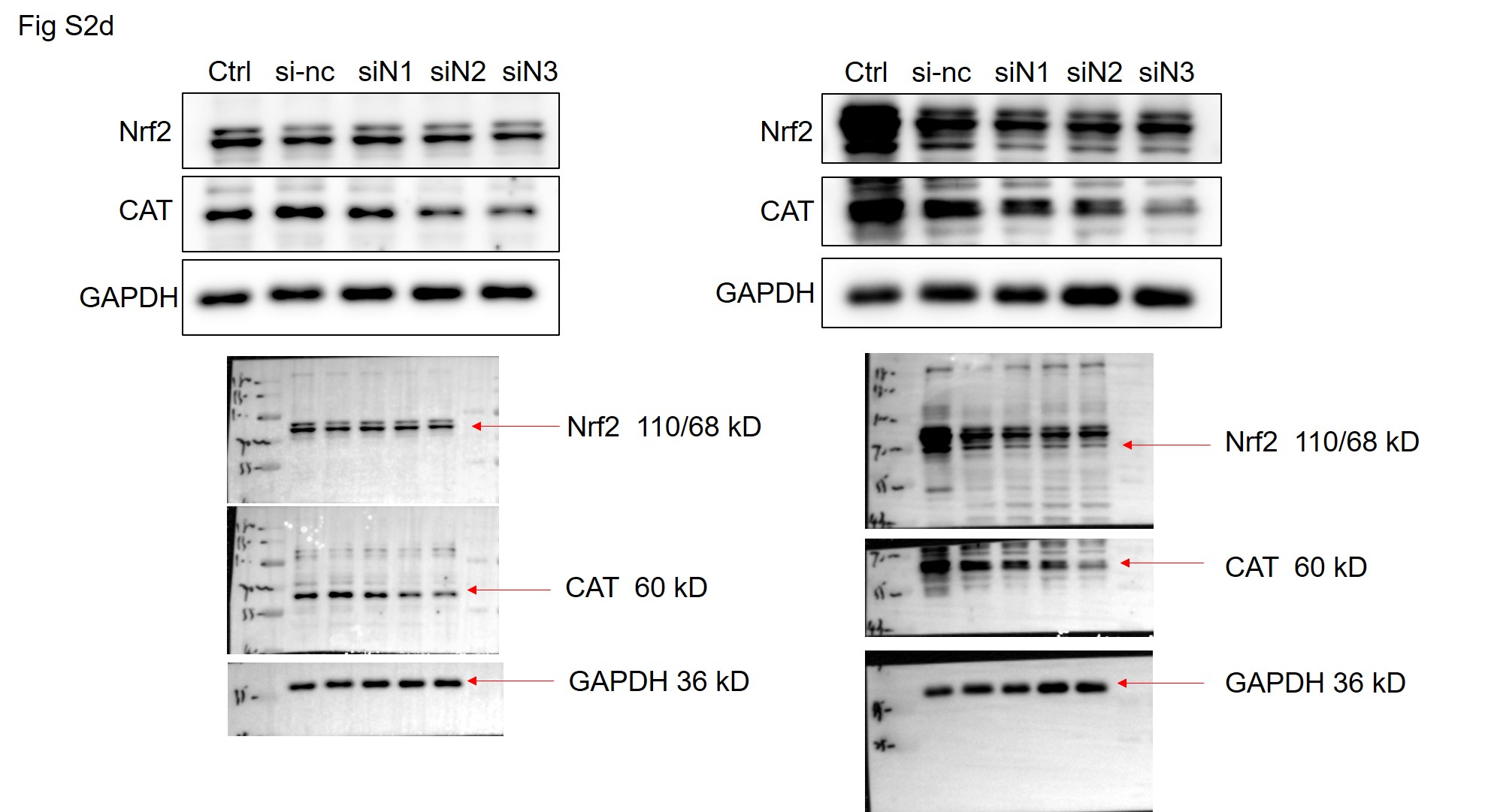

Supplement: Supplementary file 5 — Supplementary Data 2 [file 42003_2023_4473_MOESM5_ESM.zip › Supplementary Data 2/Fig. S2d.jpg]

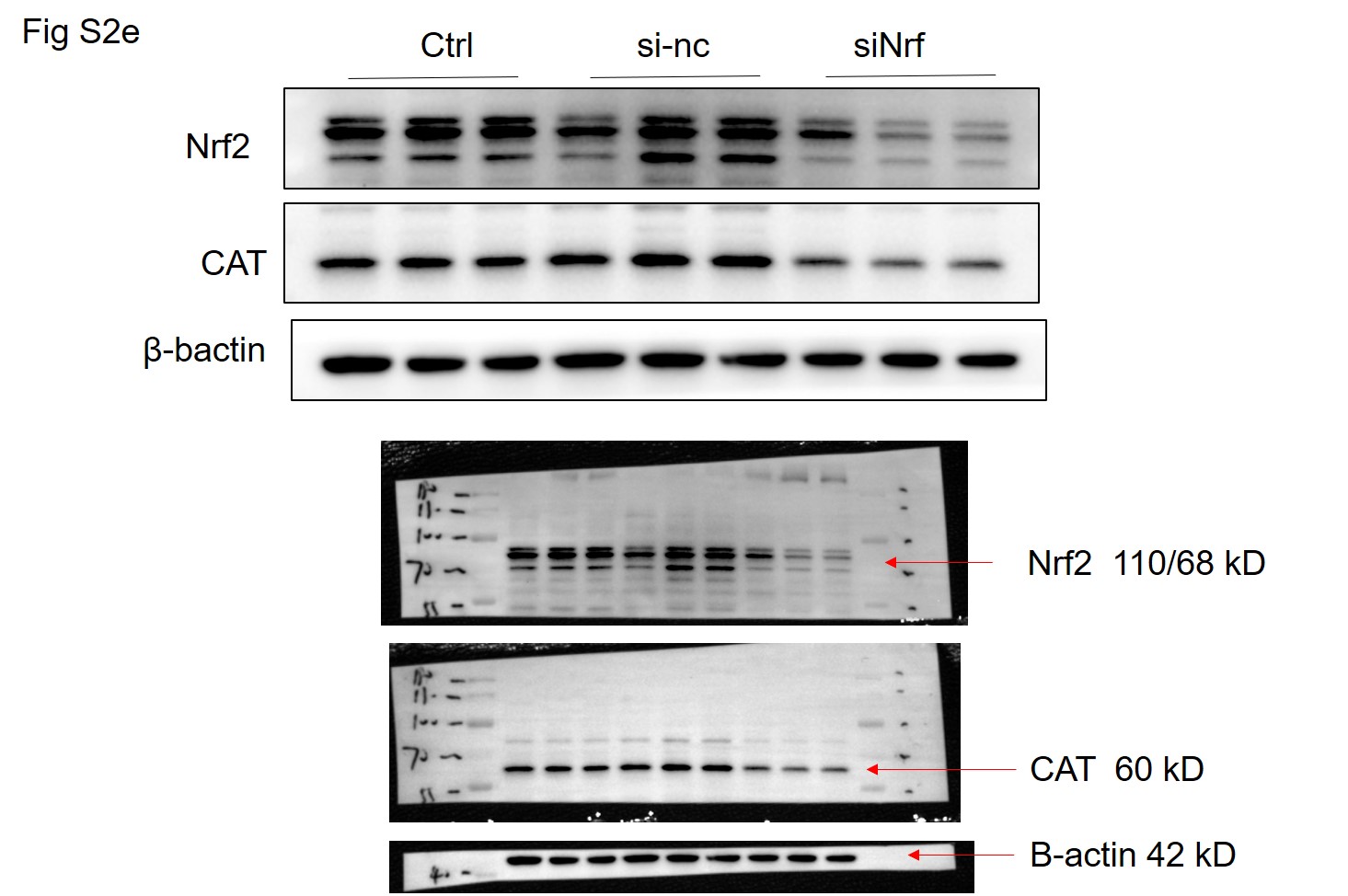

Supplement: Supplementary file 5 — Supplementary Data 2 [file 42003_2023_4473_MOESM5_ESM.zip › Supplementary Data 2/Fig. S2e.jpg]

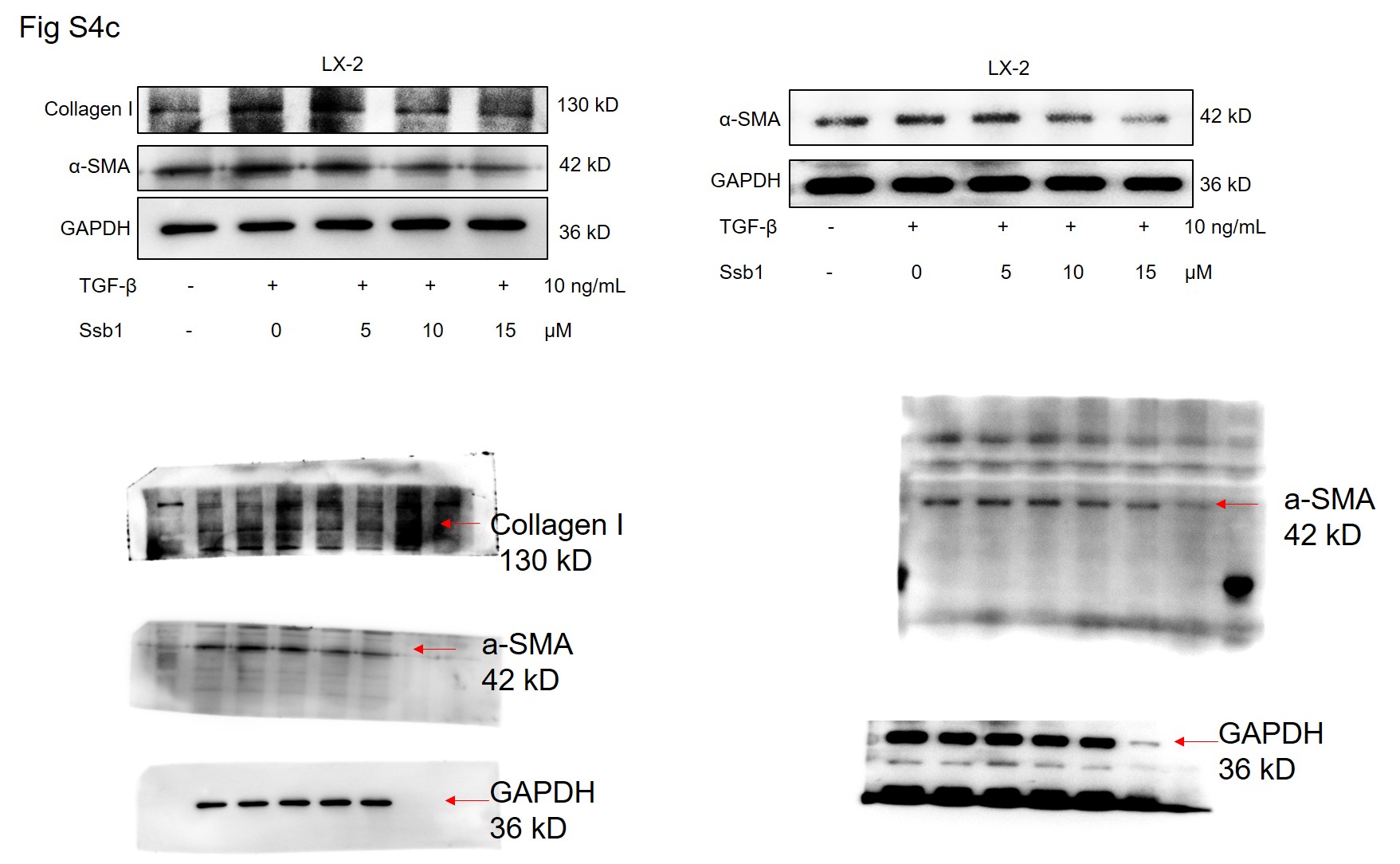

Supplement: Supplementary file 5 — Supplementary Data 2 [file 42003_2023_4473_MOESM5_ESM.zip › Supplementary Data 2/Fig. S4c.jpg]

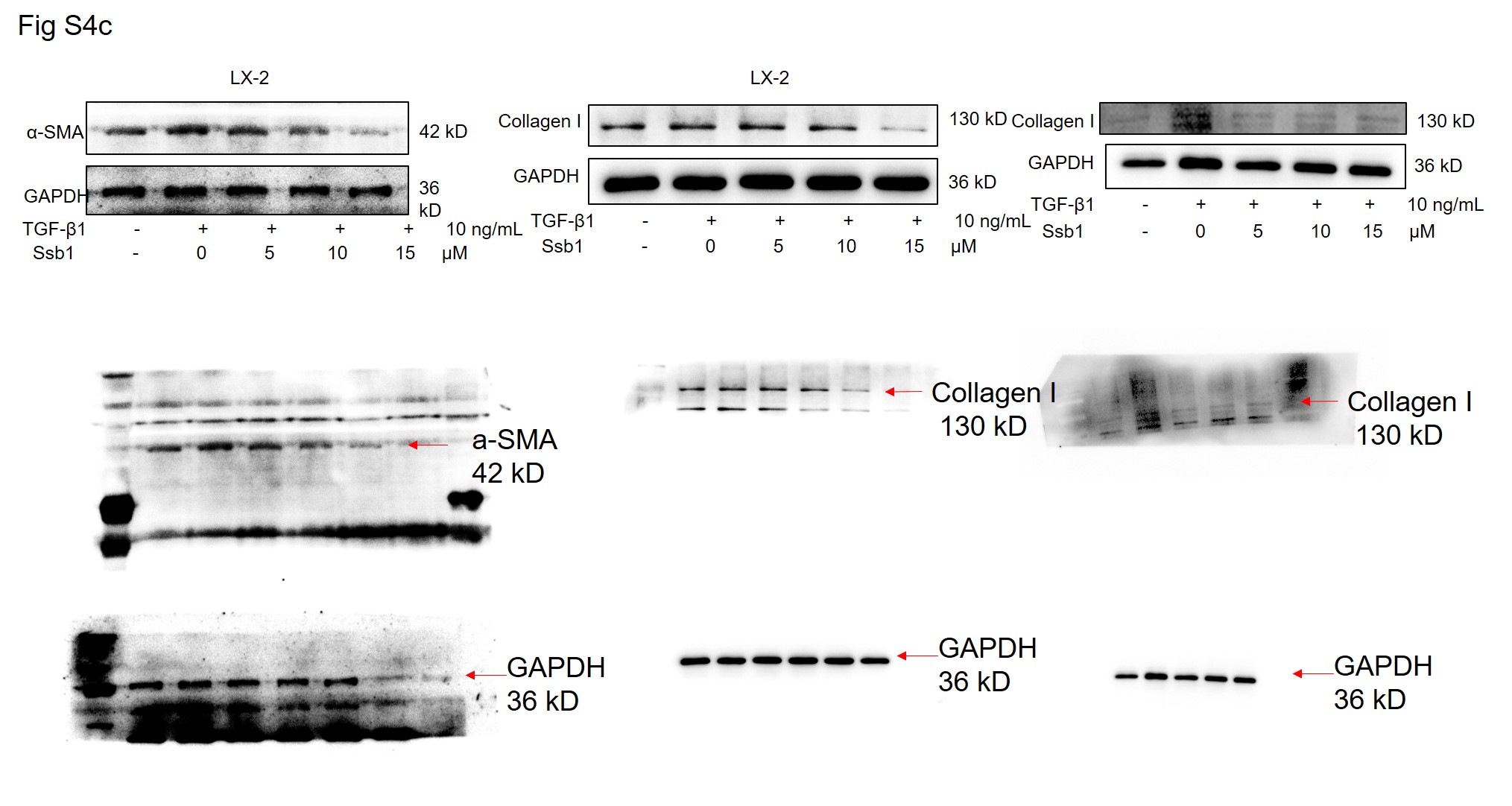

Supplement: Supplementary file 5 — Supplementary Data 2 [file 42003_2023_4473_MOESM5_ESM.zip › Supplementary Data 2/Fig. S4c-1.jpg]

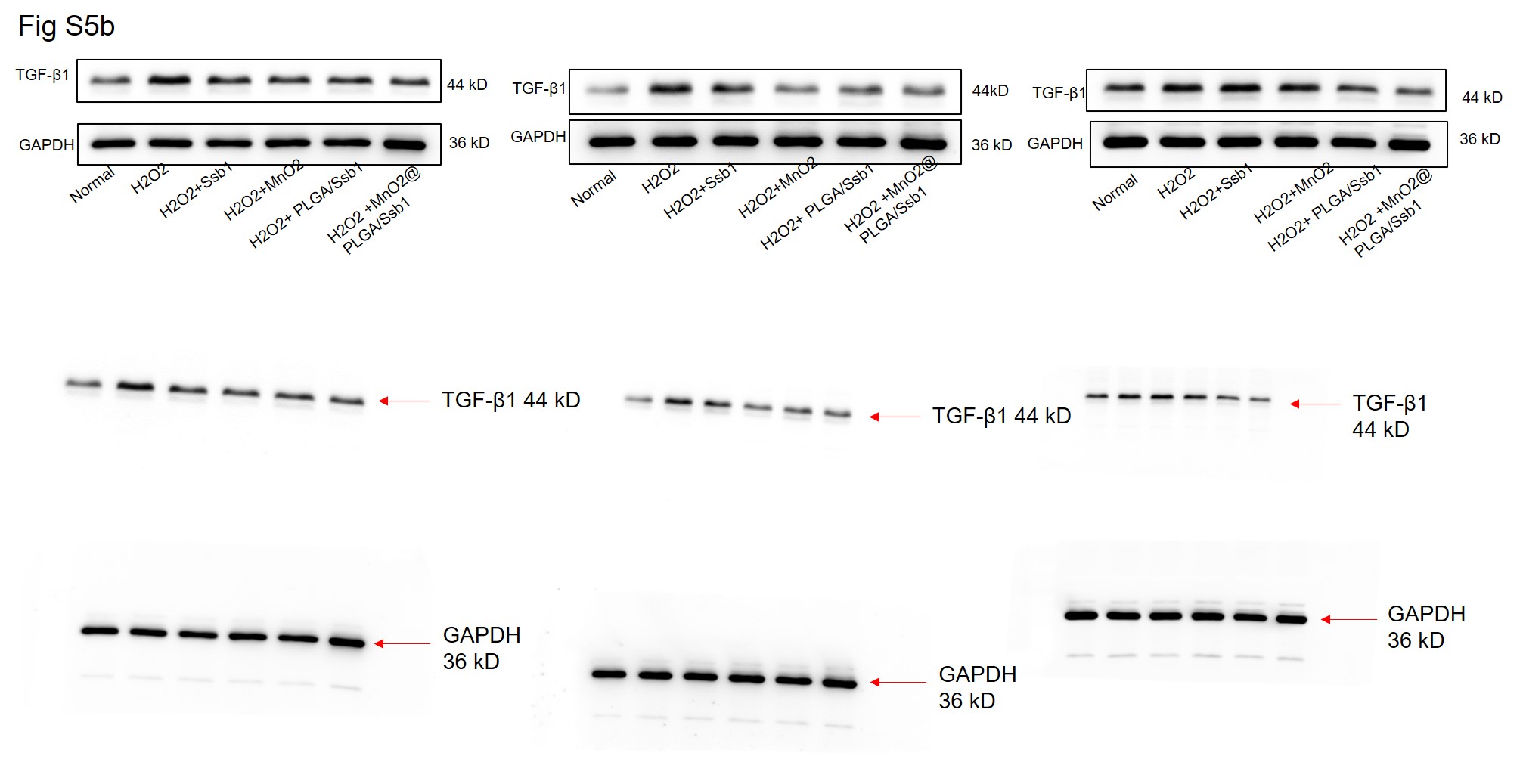

Supplement: Supplementary file 5 — Supplementary Data 2 [file 42003_2023_4473_MOESM5_ESM.zip › Supplementary Data 2/Fig. S5b.jpg]

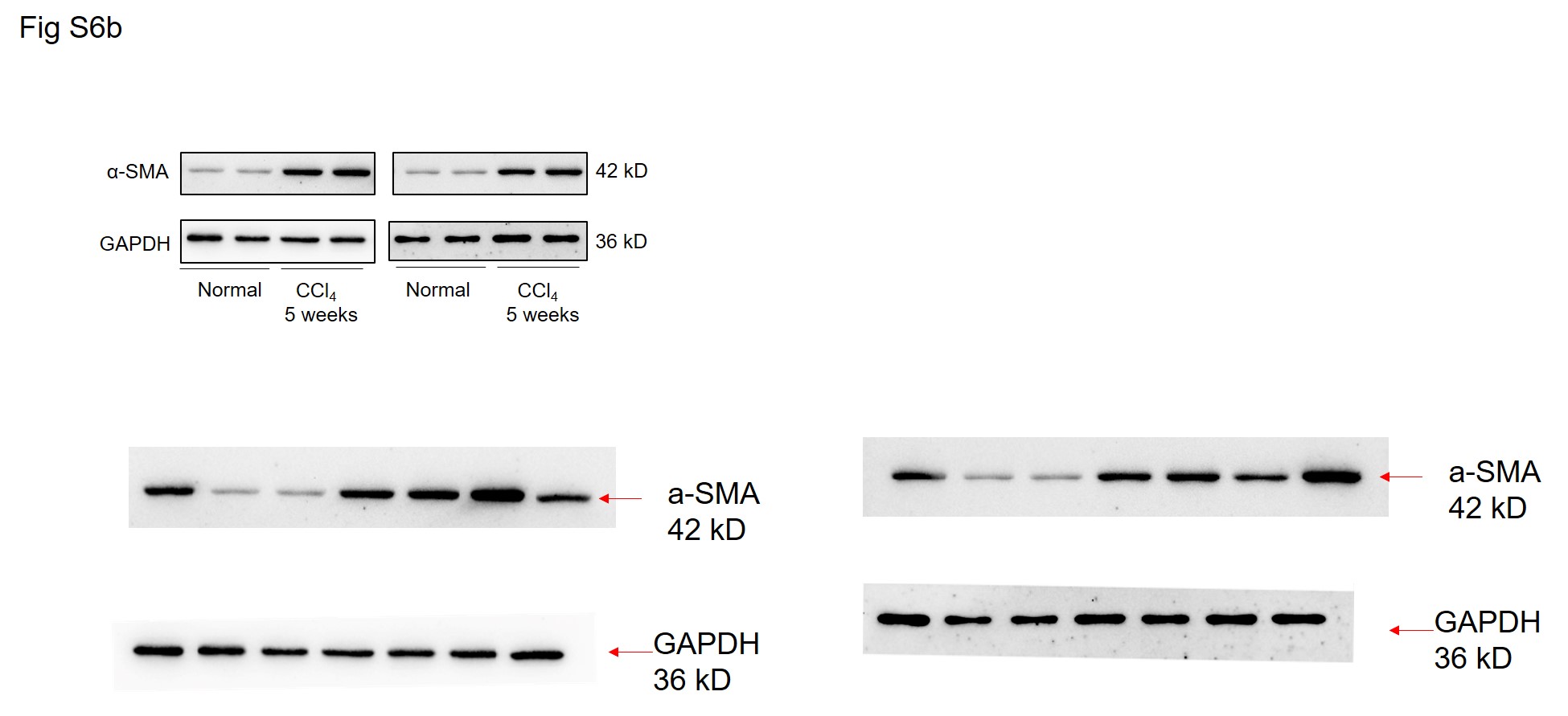

Supplement: Supplementary file 5 — Supplementary Data 2 [file 42003_2023_4473_MOESM5_ESM.zip › Supplementary Data 2/Fig. S6b.jpg]

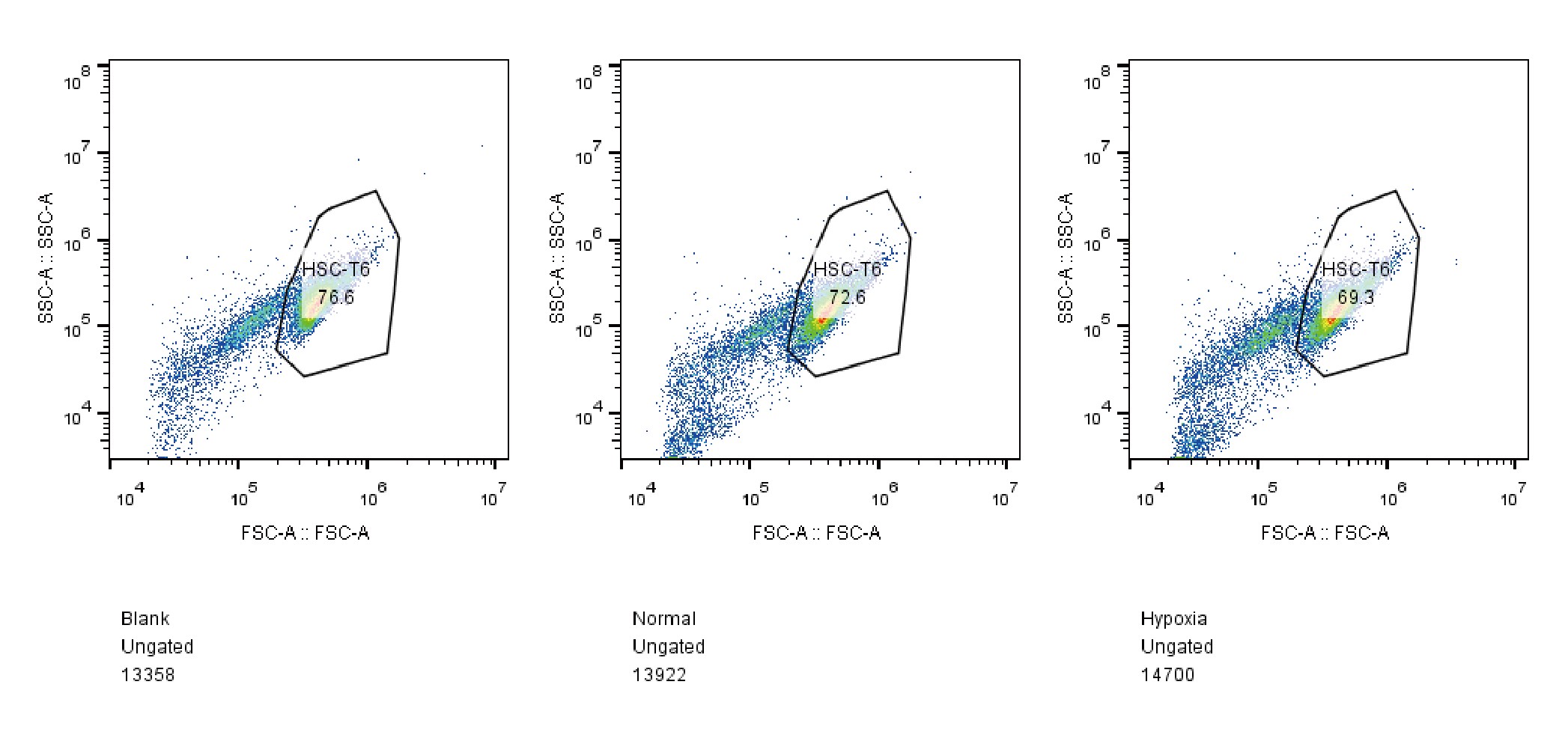

Supplement: Supplementary file 6 — Supplementary Data 3 [file 42003_2023_4473_MOESM6_ESM.zip › Supplementary Data 3/FCM gate of Figure 2h.jpg]

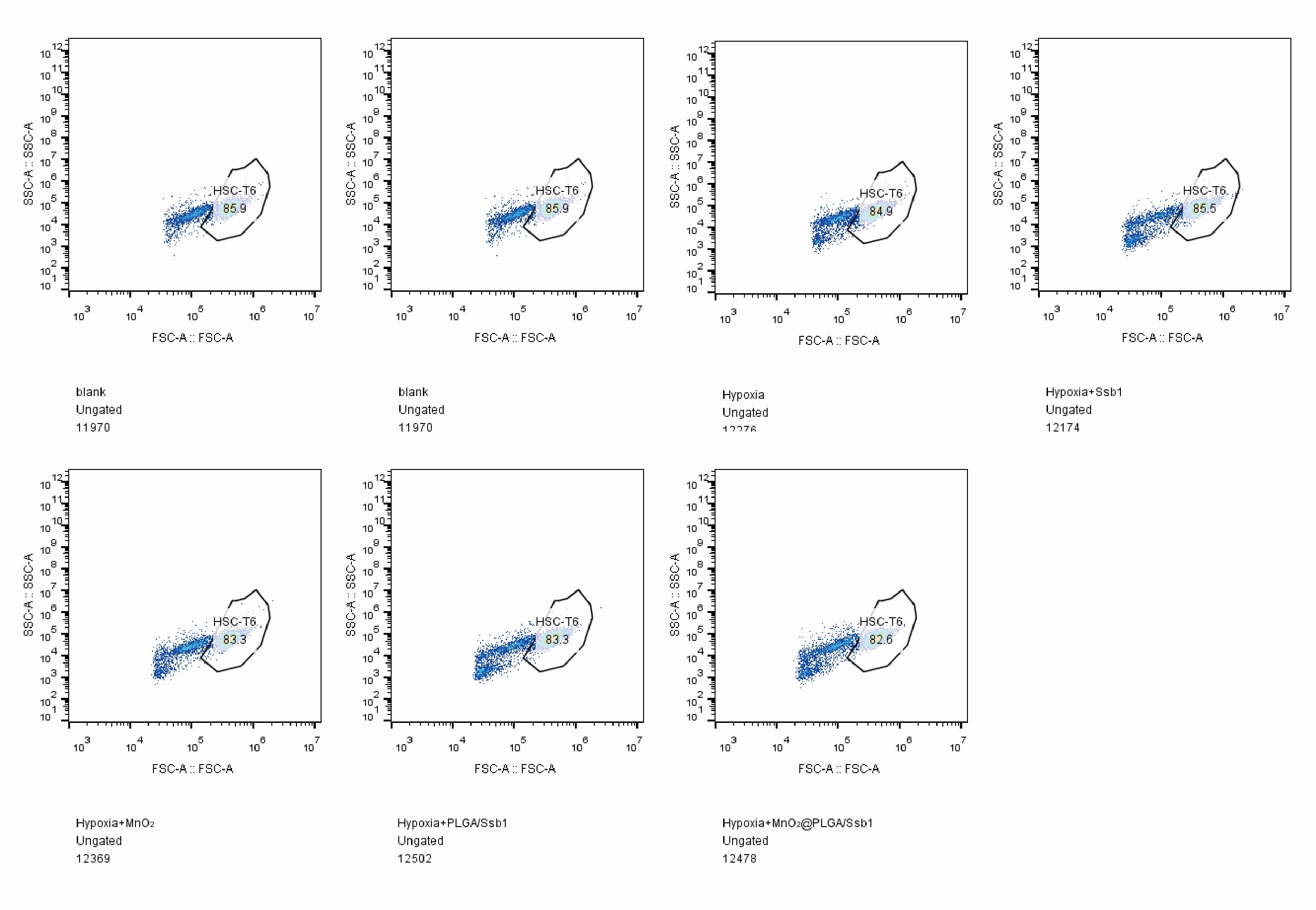

Supplement: Supplementary file 6 — Supplementary Data 3 [file 42003_2023_4473_MOESM6_ESM.zip › Supplementary Data 3/FCM gate of Supplementary Figure 5a.jpg]
