# Supplementary material for: Nanodrug rescues liver fibrosis via synergistic therapy with H2O2 depletion and Saikosaponin b1 sustained release
Source: Commun Biol. 2023 Feb 16;6:184. doi: 10.1038/s42003-023-04473-2 (PMC9935535; doi:10.1038/s42003-023-04473-2)
Supplement: Supplementary file 7 — Reporting Summary [file 42003_2023_4473_MOESM7_ESM.pdf]

nature portfolio

Corresponding author(s): Mengyun Peng, Gang Cao

Last updated by author(s): Dec 28, 2022

Reporting Summary

Nature Portfolio wishes to improve the reproducibility of the work that we publish. This form provides structure for consistency and transparency in reporting. For further information on Nature Portfolio policies, see our Editorial Policies and the Editorial Policy Checklist.

Statistics

For all statistical analyses, confirm that the following items are present in the figure legend, table legend, main text, or Methods section.

n/a	Confirmed	
☐	☒	The exact sample size (*n*) for each experimental group/condition, given as a discrete number and unit of measurement
☐	☒	A statement on whether measurements were taken from distinct samples or whether the same sample was measured repeatedly
☐	☒	The statistical test(s) used AND whether they are one- or two-sided<br>*Only common tests should be described solely by name; describe more complex techniques in the Methods section.*
☐	☒	A description of all covariates tested
☐	☒	A description of any assumptions or corrections, such as tests of normality and adjustment for multiple comparisons
☐	☒	A full description of the statistical parameters including central tendency (e.g. means) or other basic estimates (e.g. regression coefficient) AND variation (e.g. standard deviation) or associated estimates of uncertainty (e.g. confidence intervals)
☐	☒	For null hypothesis testing, the test statistic (e.g. *F*, *t*, *r*) with confidence intervals, effect sizes, degrees of freedom and *P* value noted<br>*Give P values as exact values whenever suitable.*
☐	☒	For Bayesian analysis, information on the choice of priors and Markov chain Monte Carlo settings
☐	☒	For hierarchical and complex designs, identification of the appropriate level for tests and full reporting of outcomes
☐	☒	Estimates of effect sizes (e.g. Cohen's *d*, Pearson's *r*), indicating how they were calculated

Our web collection on statistics for biologists contains articles on many of the points above.

Software and code

Policy information about availability of computer code

Data collection	*Provide a description of all commercial, open source and custom code used to collect the data in this study, specifying the version used OR state that no software was used.*
Data analysis	*Provide a description of all commercial, open source and custom code used to analyse the data in this study, specifying the version used OR state that no software was used.*

For manuscripts utilizing custom algorithms or software that are central to the research but not yet described in published literature, software must be made available to editors and reviewers. We strongly encourage code deposition in a community repository (e.g. GitHub). See the Nature Portfolio guidelines for submitting code & software for further information.

Data

Policy information about availability of data

All manuscripts must include a data availability statement. This statement should provide the following information, where applicable:

- Accession codes, unique identifiers, or web links for publicly available datasets
- A description of any restrictions on data availability
- For clinical datasets or third party data, please ensure that the statement adheres to our policy

All data supporting the findings of this study are available within the article and its supplementary information files or from the corresponding author upon reasonable request. The transcriptome sequence data have been submitted to the Sequence Read Archive (SRA) databases under BioProject number PRJNA916271.

Human research participants

Policy information about studies involving human research participants and Sex and Gender in Research.

Reporting on sex and gender	Sex and gender are not collected because the cirrhosis samples are rare and hard to collected.
Population characteristics	See above
Recruitment	Paraffin-embedded human liver tissues from three patients were collected.
Ethics oversight	Approved by Ethics Committee of the First Affiliated Hospital of Guizhou University of Traditional Chinese Medicine (Project no. K2021-066).

Note that full information on the approval of the study protocol must also be provided in the manuscript.

Field-specific reporting

Please select the one below that is the best fit for your research. If you are not sure, read the appropriate sections before making your selection.

☒ Life sciences ☐ Behavioural & social sciences ☐ Ecological, evolutionary & environmental sciences

For a reference copy of the document with all sections, see nature.com/documents/nr-reporting-summary-flat.pdf

Life sciences study design

All studies must disclose on these points even when the disclosure is negative.

Sample size	OR
Data exclusions	OR
Replication	OR
Randomization	OR
Blinding	OR

Reporting for specific materials, systems and methods

We require information from authors about some types of materials, experimental systems and methods used in many studies. Here, indicate whether each material, system or method listed is relevant to your study. If you are not sure if a list item applies to your research, read the appropriate section before selecting a response.

Materials & experimental systems

n/a	Involved in the study	
☐	☒	Antibodies
☐	☒	Eukaryotic cell lines
☒	☐	Palaeontology and archaeology
☐	☒	Animals and other organisms
☒	☐	Clinical data
☒	☐	Dual use research of concern

Methods

n/a	Involved in the study	
☒	☐	ChIP-seq
☐	☒	Flow cytometry
☒	☐	MRI-based neuroimaging

Antibodies

Antibodies used	a-SMA (14395-1-AP, Proteintech), Catalase (21260-1-AP, Proteintech), HIF-1α (AF1009, Affinity Biosciences), GAPDH (AF1186, Beyotime), TGF-β1 (ab215715, Abcam), Collagen I (14695-1-AP, Proteintech), Caspase-3 p12 (ab179517, Abcam), α-Tubulin (11224-1-AP, Proteintech), α-SMA (19245, Cell Signaling Technology), HIF-1α (cs36169, Cell Signaling Technology) and Catalase (66765-1-Ig, Proteintech), Foxo3a (AF7624, Affinity), Nrf2 (16396-1-AP, Proteintech), β-actin (66009-1-Ig, Proteintech)
Validation	a-SMA (14395-1-AP) apply in FC, IF, IHC, IP, WB, ELISA and shows reactivity with human, mouse, rat samples.<br>Catalase (21260-1-AP) apply in IF, IHC, WB, ELISA and shows reactivity with human, mouse, rat samples.

HIF-1α (AF1009) applies in WB, IHC, IF/ICC, ELISA(peptide) and shows reactivity with human, mouse, rat samples.
GAPDH (AF1186) applies in WB, IP, IF, IHC, ICC, FC and shows reactivity with human, mouse, rat samples.
TGF-β1 (ab215715) applies in WB, IHC-P and shows reactivity with mouse, rat, human samples.
Collagen Type I (14695-1-AP) applies in WB, IP, IHC, IF, FC, ELISA applications and shows reactivity with mouse, human, pig, rat samples.
Caspase-3 p12 (ab179517) applies in WB, IHC-P, ICC/IF, Flow Cyt (Intra) and shows reactivity with mouse, rat, human samples.
α-Tubulin (11224-1-AP) applies in WB, IP, IHC, IF, FC, CoIP, ELISA and shows reactivity with human, mouse, rat samples.
α-SMA (19245) applies in WB, IP,IHC, IF and shows reactivity with human, mouse, rat samples.
HIF-1α (36169) applies in WB, IP, IF, F,ChIP, C&R and shows reactivity with human, mouse, monkey samples.
Catalase (66765-1-Ig) applies in WB, IHC, IF, ELISA and shows reactivity with Human, Mouse, Rat, Pig samples.
Foxo3a (AF7624) applies in WB, IF/ICC, ELISA (peptide) and shows reactivity with Human, Mouse, Rat samples.
Nrf2 (16396-1-AP) applies in FC, IF, IHC, IP, WB, CoIP, ChIP, ELISA and shows reactivity with Human, Mouse, Rat samples.
β-actin (66009-1-Ig) applies in FC, IF, IHC, IP, WB, ELISA and shows Human, Mouse, Rat, Hamster, Monkey, Dog, Pig, Chicken, Rabbit, Zebrafish samples.

Eukaryotic cell lines

Policy information about cell lines and Sex and Gender in Research

Cell line source(s)	Human hepatic stellate cell line LX-2 was purchased from Procell Life Science & Technology Co., Ltd. (Shanghai, China). Rat hepatic stellate cell line HSC-T6 was provided generously by Prof. Su Tao (Guangzhou University of Chinese Medicine).
Authentication	OR
Mycoplasma contamination	OR
Commonly misidentified lines (See ICLAC register)	*Name any commonly misidentified cell lines used in the study and provide a rationale for their use.*

Animals and other research organisms

Policy information about studies involving animals; ARRIVE guidelines recommended for reporting animal research, and Sex and Gender in Research

Laboratory animals	SPF Balb/c mice
Wild animals	*Provide details on animals observed in or captured in the field; report species and age where possible. Describe how animals were caught and transported and what happened to captive animals after the study (if killed, explain why and describe method; if released, say where and when) OR state that the study did not involve wild animals.*
Reporting on sex	male
Field-collected samples	OR
Ethics oversight	All experiments of animals have received the approval of Ethics Committee of Zhejiang Chinese Medical University (Hangzhou, China, Project no. SYXK[illegible]2021-0012) and all 309 animals have received good care.

Note that full information on the approval of the study protocol must also be provided in the manuscript.

Flow Cytometry

Plots

Confirm that:

- [x] The axis labels state the marker and fluorochrome used (e.g. CD4-FITC).
- [x] The axis scales are clearly visible. Include numbers along axes only for bottom left plot of group (a 'group' is an analysis of identical markers).
- [ ] All plots are contour plots with outliers or pseudocolor plots.
- [x] A numerical value for number of cells or percentage (with statistics) is provided.

Methodology

Sample preparation	Rat liver stellate cell line HSC-T6 at a density of 5 X 105 cells/well in a 6-well cell culture plate were exposed to normoxia and hypoxia (5 % O2) with different treatment. 24 hours later, cells were incubated with 10 mM DCFH-DA.in working solution at 37 ? for 30 min in darkness. Then the medium was removed and HSC cells were washed with PBS for three times. Fluorescent signals were analyzed by CytoFlex flow cytometry (Beckmancoulter, CytoFlex, USA).
Instrument	CytoFlex flow cytometry (Beckmancoulter, CytoFlex, USA)

Software	FlowJo v10
Cell population abundance	10000 cells
Gating strategy	FSC/SSC

☒ Tick this box to confirm that a figure exemplifying the gating strategy is provided in the Supplementary Information.
