# Supplementary material for: Nanodrug rescues liver fibrosis via synergistic therapy with H2O2 depletion and Saikosaponin b1 sustained release
Source: Commun Biol. 2023 Feb 16;6:184. doi: 10.1038/s42003-023-04473-2 (PMC9935535; doi:10.1038/s42003-023-04473-2)
Supplement: Supplementary file 4 — Supplementary Data 1 [file 42003_2023_4473_MOESM4_ESM.zip › ▓Γ╨≥╜ß╣√/PSC115013-1.H1-F.10314006.G11536B04D12(╓╨╢╧OK).doc]

CAGAAAATCAACTAAAGATTACAAAAACAAATTACAAAAATTCAAAATTTTCGGGTTTATTACAGGGACAGCAGAGATCCAGTTTGGTTAATTAATCGAGCGGCCGCCCCCTTCACCGAGGGCCTATTTCCCATGATTCCTTCATATTTGCATATACGATACAAGGCTGTTAGAGAGATAATTGGAATTAATTTGACTGTAAACACAAAGATATTAGTACAAAATACGTGACGTAGAAAGTAATAATTTCTTGGGTAGTTTGCAGTTTTAAAATTATGTTTTAAAATGGACTATCATATGCTTACCGTAACTTGAAAGTATTTCGATTTCTTGGCTTTATATATCTTGTGGAAAGGACGAAACACCGGGGGTAAGACTACCCGCCGACCCTAGCCCGGGGTTCCGCTTCCCAAATTGAAAAAATAACTTTTGAGATTGGGAAAAACCCAACAAAAAAGGAATGAAAAAAATGCTTCTTTTGGAATTTGTTAGTGCAATATCTTTTATTGGAACATTTTAAAGGGTAAAAAAAAAATTGGGGGGGGGGGAAAAAAAATCCGAATGAAAAAACCCCCCCCTGTGGGGATAACACCCCCCCCGGGGGCCCCGGAAAAAAATTTTCAAACCCCCAACTTTTTTTTAAAAAGGGGGGCGGGGGAAAACAAAATTTCTAACACTGTGTTGTCCCTGGCTCCCCTGGCCAAAAAGTGACCCATTTTTTCGGCCTGGGTTCTCCAGGGGGAAACCCCCCCCCCCCCGCGGGGGCCCCCCCTATTGGACAGGGCGGCCCCCCGGGGGGCTCCCCAAAAATTTCGGGAAAAAAATCTAAAAACTTCGGGTAAAACTTTTTGGGCCCCCCCCCCCACCCAAGCAAAGGTGTTTCGCGCACCCCCCGGCTCTGACCCCGTTGAAAAAAGGGGGTTTTATTCCGCGAGAACCCGGGGGGATAGCCCCCTCTCCAAGTCCCACGAGAAAATAATACGGGGGGGTTGAAAAAATA

PSC115013-1	ccggGGGTAAGTCGAGAAGTGTTTGctcgagCAAACACTTCTCGACTTACCCtttttg
